# Smart biomaterials in restorative dentistry: Recent advances and future perspectives

**DOI:** 10.1016/j.mtbio.2025.102349

**Published:** 2025-09-29

**Authors:** Jianpeng Sun, Jingang Jiang, Zhiyuan Huang, Xuefeng Ma, Tao Shen, Jie Pan, Zhuming Bi

**Affiliations:** a Key Laboratory of Advanced Manufacturing and Intelligent Technology, Ministry of Education, Harbin University of Science and Technology, Harbin, 150080, China; b Heilongjiang Province Key Laboratory of Pattern Recognition and Information Perception and Technology, Harbin, 150080, China; c Key Laboratory of Engineering Dielectrics and Its Application, Ministry of Education, Harbin University of Science and Technology, Harbin, 150080, China; d State Key Laboratory of Robotics and System, Harbin Institute of Technology, Harbin, 150090, China; e Key Lab of Structures Dynamic Behavior and Control of the Ministry of Education, Harbin Institute of Technology, Harbin, 150090, China; f Peking University School of Stomatology, Peking, 100081, China; g Purdue University Northwest, Hammond, 46323, United States of America

**Keywords:** Bioactive materials, Smart materials, Dental restorative materials, Drug delivery vehicles, Hydrogels, NACPs

## Abstract

Smart biomaterials in restorative dentistry are engineered to detect and respond to diverse physiological and environmental stimuli, including mechanical stress, pH fluctuations, temperature changes, and magnetic or electrical fields. Unlike conventional inert restorative materials, these intelligent systems integrate both diagnostic and therapeutic functions into clinical practice. This review systematically summarizes recent advances in smart restorative materials using a methodology that combines literature-based classification with comparative evaluation. The materials are categorized into nanocomposites, hydrogels, bioactive agents, chemical and resin-based systems, intelligent carrier platforms, and ceramic-based materials. For each category, we examine design strategies, underlying mechanisms, and representative experimental or preclinical findings. The analysis highlights progress in drug delivery, antibacterial–remineralization coupling, self-healing capacity, and tissue engineering applications. In addition, the review outlines the advantages and limitations of each material class, evaluates their readiness for clinical translation, and identifies technical barriers to widespread adoption. Finally, future research directions are discussed, including multi-stimulus coupling, bioinspired functionalization, and integration with digital dentistry technologies. Overall, this review provides a comprehensive methodological and developmental perspective on smart restorative materials, with the goal of supporting their translation from laboratory innovation to clinical application.

## Graphical abstract

**Figure:**
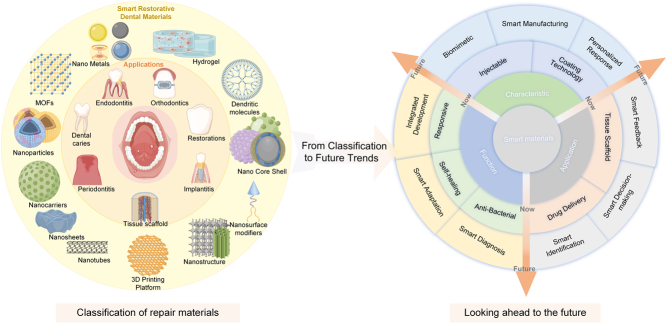

## 1 Introduction

Teeth possess limited regenerative capacity compared with other tissues, which makes dental materials indispensable for restoring normal function [1]. By 2021, major oral diseases such as untreated dental caries, severe periodontitis, edentulism, and related conditions had affected approximately 3.69 billion people worldwide. Among these, untreated caries in permanent teeth represented the leading global burden of disease [2]. A study conducted in Guangxi, China, reported that fluoride coatings combined with oral health education interventions cost USD 25.36 per case of caries prevented [3]. The high prevalence of caries and the substantial treatment costs underscore the urgent need for smart restorative materials. These materials not only adapt to complex oral conditions, such as acidic environments and masticatory stress, but also enhance antimicrobial properties, reduce the risk of secondary caries, and extend the service life of restorations. As technology advances, smart restorative materials enable personalized treatment by responding to environmental stimuli such as temperature and pH changes, thereby lowering treatment costs, minimizing recurrence, and improving oral health.

Restorative materials are widely used to treat pathological dental conditions. Although daily oral care and improved awareness can reduce the incidence of caries, periodontitis, pulpitis, and fissures, many individuals neglect preventive measures until symptoms appear, leading to persistently high disease rates. With the development of modern dentistry, the demand for diverse restorative materials has increased across multiple specialties, including periodontology and implantology. The performance of restorative materials directly determines the success of restorations; however, the oral environment is highly complex, and conventional materials sometimes fail to meet clinical requirements [4]. For instance, bonding agents are susceptible to hydrolytic degradation, resulting in secondary caries, while long-term cyclic loading can induce fatigue cracks in restorations. Consequently, ideal restorative materials must withstand chemical challenges from food as well as mechanical stresses from mastication. Traditional restorative features are no longer sufficient. With advances in technology and fabrication methods, dental materials are being enhanced with “smart” properties, such as superior mechanical strength and increased antimicrobial activity [5], [6].

Smart restorative materials respond to environmental stimuli with adaptive changes in properties, such as pH [7], temperature [8], and light parameters (wavelength, intensity, etc.) [9], [10], to address specific clinical needs. On the basis of their degree of intelligence, smart biomaterials can be classified into four categories: inert, active, reactive, and autonomous [11]. Inert biomaterials exhibit minimal interaction with surrounding tissues; examples include ceramics, stainless steel, and titanium, which are commonly used in dental restorations. Active biomaterials elicit biological responses upon contact with human tissues, providing therapeutic benefits such as releasing anti-inflammatory agents under adverse conditions. Reactive biomaterials, also referred to as biosensing or stimulating materials, can detect environmental stimuli and respond to in vivo or in vitro signals. Autonomous biomaterials further adapt their functional responses dynamically, delivering context-specific effects at different stages of treatment; examples include pH-responsive hydrogels and thermosensitive drugs that liquefy only at elevated temperatures.

The boundaries between categories are not always distinct, as some materials may transition between types. For example, surface coatings or functionalization with embedded particles can transform bio-inert materials into higher-level bio-intelligent materials, illustrating the potential of cross-disciplinary approaches. Although the functions of smart restorative materials are not yet fully exploited, insights from bio-smart materials in other fields highlight promising opportunities for dental applications. However, insufficient mechanistic understanding continues to hinder their systematic classification and clinical translation.

Overall, restorative dental materials benefit substantially from intelligent functionalities, whether in hard- or soft-tissue applications. This review aims to summarize the development of representative smart restorative materials, classify them by material source and clinical application, clarify their restorative mechanisms, and analyze their advantages and limitations. Finally, future development trends are discussed to provide guidance for the clinical translation of smart restorative materials in dentistry and related fields.

## 2 Clinical challenges and drawbacks of the current restorative dental materials

The primary function of dental restorative materials is to re-establish normal masticatory function, enabling patients to eat properly and meet daily nutritional needs. This fundamental role has guided the development of restorative materials. Their essential functions include the adhesion of bonding agents, the mechanical properties of restorations (e.g., inlays and crowns), and the anti-degradation properties and biocompatibility of cement materials. These traditional materials provide the foundation for the development of smart dental materials, as improvements in mechanical, chemical, and biological properties are crucial for the effectiveness of next-generation intelligent systems.

With rising living standards and advances in technology, patient needs have evolved, generating demand for restorative materials with intelligent functionalities. Currently, commercially available materials cannot fully meet these diverse requirements. However, sustained research has led to the development of several laboratory materials that demonstrate promising performance. Although these materials remain in preclinical stages due to concerns about stability and safety, they are steadily progressing toward commercialization and may eventually be adopted in clinical practice.

Smart restorative materials retain the basic functions of conventional systems while offering additional capabilities, such as antibacterial properties or tissue-regenerative potential. They can also address clinical challenges proactively by responding to external stimuli and adapting to environmental conditions. For example, such materials may release drugs or eliminate cariogenic bacteria in response to disease progression, thereby enhancing oral health. Despite this potential, current restorative materials still face limitations due to insufficient intelligence, highlighting the need for continued improvement.

Conventional restorative materials may also cause adverse reactions. For instance, poly(methyl methacrylate) can retain unreacted monomers during curing, which may leach into the oral cavity and trigger allergic responses such as contact mucositis, burning sensations, or xerostomia [12]. Nickel–titanium alloys used in orthodontics contain over 50% nickel, releasing ions at levels sufficient to induce allergic reactions [13]. While fluoride remains an effective agent for caries prevention, high doses or frequent exposure may cause local mucosal irritation, including rashes, tingling, or mottled enamel. Allergic reactions to silicone rubber, such as contact dermatitis, have also been reported [14], as well as hypersensitivity to cobalt and chromium, which can lead to oral mucosal inflammation [15].

This review therefore examines the limitations of conventional restorative materials, categorizing them according to antimicrobial properties, biocompatibility, and resistance to degradation, in order to underscore the necessity of intelligent advancements in dental restorative materials.

### 2.1 Antimicrobial property

The oral cavity is the second most complex microbial ecosystem in the human body, comprising bacteria, viruses, fungi, and protozoa [16]. Disruption of this microbiota equilibrium can cause harmful alterations, leading to oral diseases such as dental caries, periodontitis, root canal infections, peri-implantitis, and pulpitis [17]. The oral microbiota includes more than 700 bacterial species that colonize teeth, gingiva, and oral mucosal tissues in the form of biofilms, or exist in the planktonic phase of saliva. Together with the host immune system, saliva, and local inflammatory responses, the oral microbiota maintains homeostasis in the oral environment. In healthy conditions, the oral microbiota exhibits high diversity, and a well-structured microbial community not only prevents the overgrowth of pathogenic microorganisms but also supports both oral and systemic health [18], [19]. If bacterial infections remain uncontrolled, they can progress into chronic conditions, damaging oral tissues and causing defects [20]. Secondary caries, for instance, is one of the most common complications after dental restoration, with the detection rate of *Streptococcus mutans* serving as a key indicator of caries activity [21]. Contributing factors include microleakage between the restoration and tooth, which facilitates bacterial biofilm formation, and the absence or insufficiency of antimicrobial properties in restorative materials [22]. The bonding interface between restoration and tooth often harbors bacteria that cannot be eliminated by conventional methods, leading to premature restoration failure [23]. Therefore, the development of restorative materials capable of maintaining a balanced oral microenvironment and providing long-term antimicrobial activity is crucial to reducing caries incidence. However, most current restorative materials lack sustained antimicrobial function, underscoring the need for bio-intelligent strategies.

Biofilm formation is a key mechanism of plaque accumulation and caries development. *Streptococcus mutans* promotes enamel demineralization and caries by adhering to the tooth surface, synthesizing extracellular polysaccharides, and generating an acidic microenvironment.

To counter these mechanisms, anti-biofilm material design should focus on inhibiting initial bacterial adhesion, interfering with extracellular polysaccharides synthesis, blocking quorum sensing, providing contact-based biocidal effects, or enabling drug-release strategies. Several studies have shown that tea polyphenols significantly inhibit *S. mutans* adhesion and extracellular polysaccharides synthesis, downregulate gene expression, and block glucosyltransferase function, thereby reducing biofilm formation [24], [25]. Curcumin has been reported to inhibit bacterial adhesion and extracellular polysaccharides production, as well as interfere with quorum sensing, reducing biofilm stability [24], [26]. Fucoidan sulfate polysaccharides at concentrations ≥250,μg/mL achieved complete inhibition of biofilm and colony growth of *S. mutans* and *S. sobrinus* for up to 48 h [27]. In addition, citronella essential oil suppressed *S. mutans* biofilm growth by 93% at only 1μg/mL and achieved >95% inhibition of established biofilms [28].

### 2.2 Biocompatibility

Early dental restorative designs primarily emphasized functional restoration, focusing on the mechanical, chemical, and physical properties of dental materials. These materials were selected to provide sufficient strength and maintain the structural integrity of restorations, often without considering biological interactions between dental materials and oral tissues. However, advances in biological and material sciences have demonstrated that even optimal mechanical, chemical, and physical properties are insufficient if materials harm oral tissues or pose health risks. The concept of biocompatibility was first introduced by Homsy and colleagues [29], and later defined by Williams [30] as follows: “a biomaterial should perform its intended function in a medical context without inducing harmful local or systemic effects”. Moreover, it should promote the most favorable cellular or tissue response within its specific context, thereby optimizing clinically relevant outcomes. Consequently, biocompatibility is a fundamental criterion for ensuring the safety of biomaterials and medical devices.

Nevertheless, patients often equate “safety” with the complete absence of harm. Most dental materials, classified as medical devices, come into direct contact with both hard and soft oral tissues, with some remaining embedded for extended periods. Clinical concerns more commonly involve localized adverse effects, such as direct or indirect damage to enamel, dentin, pulp, or soft tissues. Disturbances in oral flora can further disrupt ecological balance. Research on dental material biocompatibility also faces challenges, including metal contamination and uncertainties regarding the safety of nanomaterials. Despite scientific progress, many conventional dental materials still pose potential risks, underscoring the need for improvements and the integration of biocompatible intelligent materials.

Furthermore, evaluation methods for biocompatibility are evolving. Recent studies employing zebrafish embryos as an in vivo platform have revealed concentration-dependent biocompatibility profiles of commercial materials such as MTA and Biodentine. These findings connect biological effects with measurable oxidative stress, apoptosis, and specific molecular pathways [31], highlighting both the persistent safety challenges and the development of advanced tools to address them.

### 2.3 Clinical biocompatibility assessment

This section systematically elaborates on experimental approaches, common findings, and extrapolative limitations along two axes, namely evaluation methods (in vitro and in vivo) and clinical implications, to clarify how current research informs assessments of clinical safety.

In vitro assessments typically examine cytotoxicity, inflammatory responses, and cellular functions using either material extracts or direct contact tests. Commonly employed assays include cell viability (MTT, CCK-8), membrane integrity (LDH release), genotoxicity (Comet, Ames), inflammatory mediator expression (ELISA/qPCR for IL-1β, IL-6, TNF-α), oxidative stress, and differentiation or mineralization markers (ALP activity, Alizarin Red staining). Oral-specific assays often address biofilm formation and bacterial adhesion to evaluate microbial ecological impacts. The strengths of in vitro studies include high controllability and feasibility for mechanistic exploration; however, a major limitation is their inability to replicate the complex biological–chemical–physical environment of the oral cavity (e.g., saliva composition, mechanical loading, diverse microbiota, and host immunity). Thus, while positive in vitro findings may indicate potential risks, they are insufficient for predicting clinical outcomes and must be interpreted in conjunction with subsequent in vivo and clinical evidence.

In vivo studies employ animal models to assess tissue-level responses to materials. Common approaches include subcutaneous or intramuscular implantation for local inflammation and capsule formation, pulp or near-dentin exposure for pulpal toxicity and repair, and implant/bone integration for bone–material interactions. Primary endpoints typically involve histological scoring (e.g., inflammation severity, granulation tissue, fibrous capsule, necrosis), complemented by systemic biochemical analyses and organ histopathology to detect potential distal toxicity or cumulative effects. Compared with in vitro studies, in vivo models more closely approximate physiological conditions; however, limitations related to interspecies differences, dose–time scaling, and ethical considerations constrain their extrapolation to human clinical contexts.

Clinically, key concerns include long-term leaching and absorption of monomers or metal ions, sensitization or allergic reactions, sustained alterations to the oral microbiome, and localized damage to adjacent tissues (enamel, dentin, pulp, soft tissues). Importantly, different experimental systems capture distinct dimensions of risk—short-term high-dose exposures in vitro, implant-based models in vivo, and chronic low-dose exposures in human populations. Therefore, clinical safety assessments should integrate a “three-tiered evidence chain” (in vitro–in vivo–clinical follow-up) with explicit reporting of exposure form, dose, and duration. Given the high heterogeneity in study design, endpoints, and reporting practices, pooled graphical summaries may be misleading. Accordingly, this review adopts a narrative synthesis emphasizing methodological frameworks and conceptual classifications, rather than direct meta-analytical visualization, to avoid overgeneralization of heterogeneous findings.

### 2.4 Anti-degradation property

Research into the biocompatibility of dental materials continues to face challenges, particularly regarding metal contamination and the safety of nanomaterials. With growing emphasis on biodegradability, the safety of several widely used materials, including amalgam, nickel-containing alloys, and dental resins, has become increasingly controversial. Silver amalgam holds a unique position, as its use in restorative dentistry dates back to the Tang Dynasty in China [32], yet mercury, a key component, is neurotoxic. Early studies suggested that mercury was released only during filling and removal, and that once solidified it remained stable. However, advances in detection technology have shown that small amounts of mercury continue to be released long after placement, a finding that remains highly debated [33]. Similarly, the safety of nickel–chromium alloys is disputed. Although nickel has been used for over 5,500 years and is a component of certain human enzymes, some nickel compounds are carcinogenic. In addition, dental resins may release bisphenol A, which exhibits weak estrogenic activity and has been shown to affect prostate weight in mice [34], [35].

In summary, conventional restorative materials still face substantial challenges, leaving considerable room for improvement. Although widely used materials perform both functionally and aesthetically, they often fail to match natural tooth structure. Their modulus of elasticity and surface hardness are typically lower than enamel, making them more susceptible to abrasion and mechanical damage [36]; their thermal expansion coefficient differs from that of teeth, rendering them vulnerable to microleakage [37]; and they lack the gradient cushioning of the natural enamel–dentin interface, which compromises mechanical articulation [38]. Furthermore, resin-based materials may release unpolymerized monomers and other degradation products, posing risks to local tissues and systemic health [39]. These shortcomings limit their integration and long-term stability with natural teeth, making perfect restoration unattainable.

**Fig. 1:**
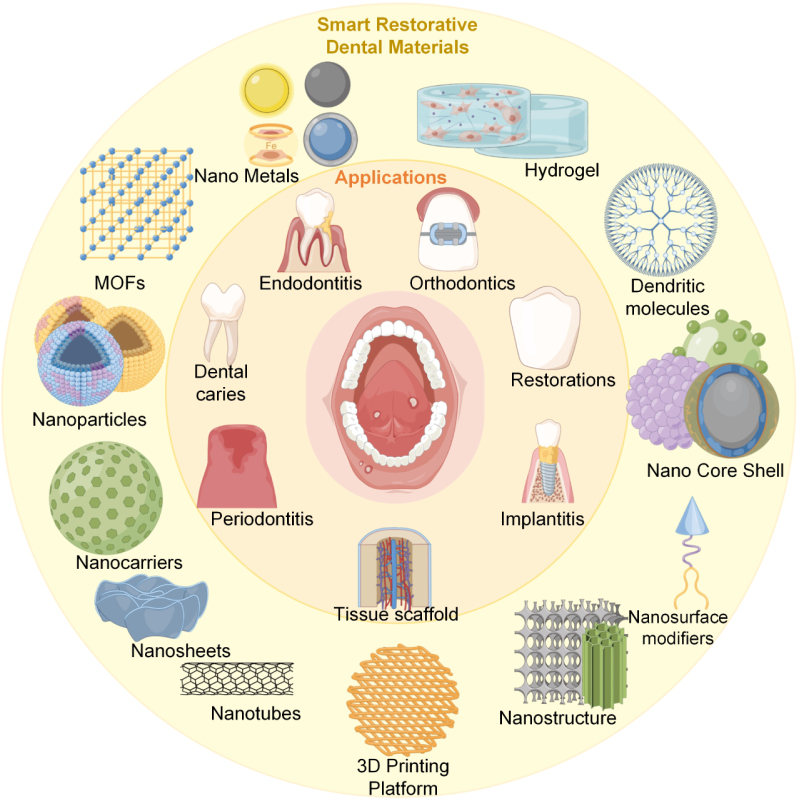
Application conditions and some material types of smart dental restorative materials.

**Fig. 2:**
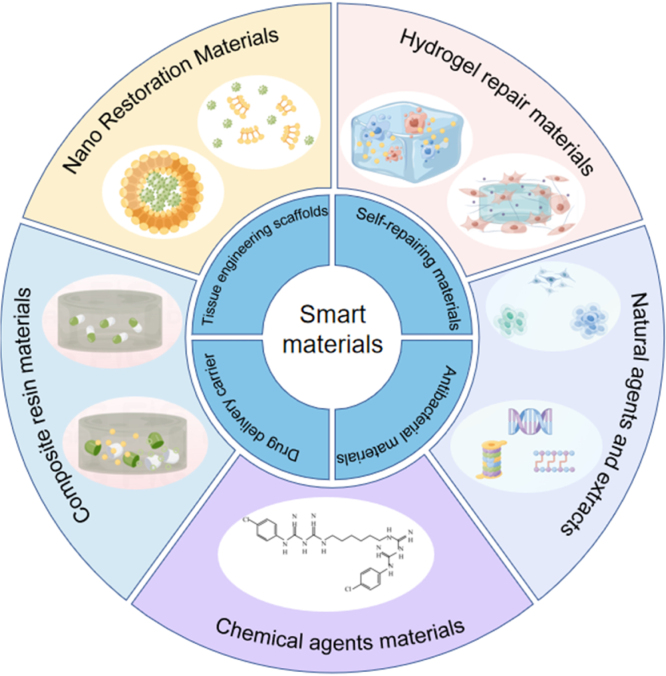
This article about the schematic structure of different smart dental restorative materials.

The performance of smart restorative materials ultimately depends on the properties of these traditional systems, which provide the structural and functional basis. However, limitations such as toxicity, biodegradability concerns, and insufficient long-term stability underscore the inability of existing materials to fully meet clinical needs. Despite rapid scientific progress, restorative materials must continue to evolve to address increasing patient demands. While newer materials are becoming more intelligent, they remain insufficient for certain conditions. Therefore, the promotion of smart restorative materials is essential for improved integration into the oral environment, alongside other intelligent materials with potential applications in dental restoration. To address current clinical challenges, researchers are developing bio-intelligent strategies derived from diverse sources, offering promising solutions to unmet clinical needs. As shown in Fig. 1, smart restorative materials encompass a broad range of applications, including drug delivery carriers, tissue engineering scaffolds, self-repairing systems, and antimicrobial agents. The overall structure of this article is outlined in Fig. 2.

## 3 Category of smart dental restorative materials

On the basis of a decade of literature review, this study classifies smart dental restorative materials into seven major categories according to their sources: nanocomposite restorative materials, hydrogel-based materials, biomaterials, chemical materials, composite resins, smart carrier systems, and ceramic or ceramic-based restorative materials. These materials encompass a wide range of oral restoration applications, including dental caries, periodontitis, endodontics, maxillofacial surgery, and other restorative fields, as shown in Fig. 1. Furthermore, by examining their application contexts, this work summarizes the scope of each material’s use and compares the relationships among different restorative materials. This comparative analysis provides valuable insights into the future development and broader adoption of restorative materials across multiple domains.

### 3.1 Nano restoration materials

**Fig. 3:**
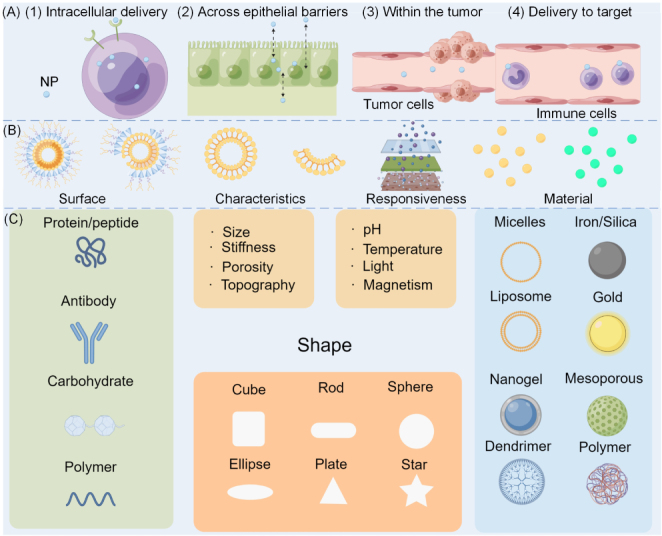
NACPs overcoming the biofilm barrier. (A) Schematic illustration of penetrating the biofilm barrier. (B) Release of NACPs in response to an external stimulus. (C) Parameters of different NACPs.

With the advancement of nanotechnology, nanomaterials are increasingly applied in dental restorative systems, demonstrating intelligent behavior through their ability to adapt to individualized conditions [40], [41]. This subsection reviews the design strategies and fabrication methods of emerging nanomaterials, emphasizing their specific functions. It establishes a technical foundation for the future development of smart nano-restorative materials in dentistry. The mechanism by which nanomaterials penetrate the biofilm barrier is illustrated in Fig. 3A and can be broadly divided into four stages. To further clarify the mechanism of nanoparticle (NACP) action, Fig. 3B presents a schematic diagram of a smart nano-carrier releasing beneficial NACPs in response to external stimuli.

#### 3.1.1 Bio-nanocomposite materials

Several factors must be carefully controlled in the application of bio-nanocomposites in the oral cavity, including controllability, reversibility, stability, antimicrobial activity, cytocompatibility, resistance to immune responses, and resistance to degradation. During drug delivery, natural bio-nanocomposites are particularly vulnerable to degradation due to their inherent instability. These challenges can be summarized as follows: (1) Natural enzymes and nanoenzymes typically exhibit high specificity toward individual reactive oxygen species but provide insufficient antioxidant protection against the diverse reactive oxygen species generated during disease progression; (2) Multi-enzyme-based antioxidant defense systems are restricted by synthetic complexity, residual by-products, and potential toxicity; (3) Natural enzymes and antioxidants generally display low stability and poor reusability, necessitating immobilization on various carriers; (4) Drug delivery systems are often difficult and costly to synthesize and may exhibit chemical or enzymatic instability, particularly when free nucleosides fail to form stable nanostructures.

To overcome these limitations, biodegradable polydopamine (PDA) NPs can be employed as smart reactive oxygen species scavengers in oxidative stress-induced periodontal disease, effectively eliminating reactive oxygen species both in vitro and in vivo [42]. PDA NPs can also serve as solvents in aqueous media for the formation of robust discrete nanoparticles [43], or as nanospheres [44] incorporated into scaffolds [45] to function as reaction carriers. For targeted applications, it is critical to select scaffolds with appropriate morphologies [46].

Small charged nanoparticles can be adsorbed onto liposomal surfaces to prevent fusion [47]. Sharp nanostructured surfaces [48] can exert bactericidal effects upon contact, offering a less invasive alternative to traditional sterilization methods. Additionally, bio-nanocomplexes have been shown to enhance the stability of dentin–composite resin interfaces [49].

One promising strategy for meeting clinical needs is the development of bio-nanocomposites that provide antimicrobial adhesion and resistance to cariogenic biofilms [50]. For instance, pH-activated nanoparticles [51], [52] enhance therapeutic resistance. Key parameters in nanoparticle carrier (NPC)–mediated drug delivery, such as diblock properties, can also be tailored to improve efficacy [53]. Moreover, nanofiber membranes can deliver antimicrobial peptides and nepheline fluorapatite glass powders [54], [55]. This novel nanotherapeutic approach holds significant potential for preventing dental caries, repairing dental defects, and controlling biofilm-associated diseases caused by pathogenic bacteria.

#### 3.1.2 Organic/inorganic nanocomposites

Organic nanocomposites are increasingly incorporated into dental restorative materials, primarily as additives to modify and enhance the properties of base materials, thereby imparting “smart” functionalities. For example, they are used in bonding agents and modified chlorhexidine (CHX) formulations to address the shortcomings of bonding agents and introduce self-healing properties. This self-healing capacity is particularly valuable for repairing the adhesive interface between dentin and restorative materials (e.g., adhesives and composite resins), thereby inhibiting microleakage and improving long-term bonding stability. In the future, this property may also play an important role in crown and indirect restorations.

Self-healing resins are typically developed using nanocapsules [56]. The effectiveness of this approach depends on nanocapsule concentration. Short-term water aging has been shown not to significantly affect either the self-healing or bactericidal performance [57]. In addition, the filler level and solution pH of nano-amorphous calcium phosphate (NACP) influence the release of calcium and phosphate ions from the binder, making NACP a representative “smart” material [58].

CHX, commonly used in oral restorations, can be “intelligentized” by incorporating various NACPs with distinct physicochemical properties, as illustrated in Fig. 3C. For instance, iron oxide (Fe${}_{3}$O${}_{4}$) NACPs [59] enable controlled release under an external magnetic field, allowing spatiotemporal regulation of drug delivery in dental resins. CHX can also be modified to be pH-responsive, facilitating release in the acidic microenvironment of cariogenic biofilms [60]. This modification reduces cytotoxicity to human oral keratinocytes compared with free CHX [61]. However, the effective delivery of antimicrobial drugs into resilient oral biofilms remains challenging due to poor penetration and insufficient pathogen targeting. Microenvironment-responsive polyethylene glycol (PEG)-shedding nanoplatforms can enhance targeted delivery into oral biofilms, thereby improving caries prevention [62]. To achieve effective local drug retention, rapid clearance from the biofilm–tooth interface must be avoided, while simultaneously ensuring microenvironment-specific targeting. Organic NACPs show great potential for multi-target binding and pH-responsive drug release [63].

Inorganic nanocomposites also play a critical role in improving the bio-intelligent performance of dental materials. Various metal-based NACPs are under investigation for this purpose. For example, incorporating ZnO NACPs into binders enhances conversion rate, flexural strength, and antimicrobial activity [64]. Additives such as aluminum oxide (Al${}_{2}$O${}_{3}$) and titanium dioxide (TiO${}_{2}$) improve wear resistance and load-bearing capacity compared to unmodified materials [65]. Bionic hybrid scaffolds with superparamagnetism have been fabricated from (  )-doped hydroxyapatite nanocrystals [66].

Magnetic hematite NACPs have been explored for managing dentin hypersensitivity [67]. Magnetite NACPs can be applied to textiles via ultrasonic methods, producing nanometallic-coated fabrics with antimicrobial activity [68]. Iron oxide NACPs (FerIONP) can also be employed to prevent dental caries [69] or encapsulated with dextran to create nanoenzymes [70], thereby broadening the functional scope of restorative materials.

Metal-based NACPs are further applied in combating healthcare-associated infections. For instance, a smart antimicrobial hybrid matrix membrane has been designed to minimize nanoparticle leaching and dispersion issues while maintaining antimicrobial efficacy [71]. Silver–metronidazole composites with nanohydroxyapatite have been developed as reinforcing fillers for periodontal pocket disinfectants [72]. Polyacid formulations with photo-reduced silver NACPs (AgNPs) exhibit strong antibacterial properties [73]. Metal NACPs are also widely used in antimicrobial photodynamic therapy (aPDT) to enhance antibacterial and anti-inflammatory effects, particularly under hypoxic conditions [74]. In such environments, Fe${}_{3}$O${}_{4}$ NACPs, chlorin e6 (a potent photosensitizer producing reactive oxygen species under specific laser wavelengths), and coumarin 6 (a hydrophobic fluorescent dye) can be encapsulated in amphiphilic silanes with conjugated structures, red-shifted absorption, and magnetic navigation properties [75]. This enables combined red- and near-infrared light stimulation for aPDT. Moreover, the limited tissue penetration of visible light in aPDT can be overcome by integrating upconversion nanomaterials responsive to near-infrared excitation [76].

Catalytic NACPs with peroxidase-like activity also serve as effective antimicrobial agents against dental plaque biofilms [77]. Mesoporous silica, a versatile coating material, further expands the functional repertoire of NACPs. For example, mesoporous silica NACPs loaded with bone-forming peptide-1 support applications in 3D bioprinting and tissue regeneration [78]. Similarly, gold nanorods benefit from mesoporous silica coatings, improving pharmaceutical loading and delivery efficiency [79]. Due to their structural robustness and high internal volume, mesoporous silica NACPs show promise for incorporating antimicrobial drugs, suggesting their integration into dental adhesives could enable responsive drug release [80].

Amorphous calcium phosphate (ACP) NACPs are widely used to enhance the “smart” behavior of restorative materials. For instance, when combined with core–shell CHX, they form CHX/ACP NACPs that serve as restorative fillers [81]. Blending ACP NACPs with dimethylaminohexadecyl methacrylate (DMAHDM) enables the development of self-healing composites capable of repairing cracks [82], [83]. Additionally, incorporating ACP NACPs with polyamidoamine or rechargeable complexes provides long-term remineralization potential [84].

Dendritic polymers, with hydrophobic cores and hydrophilic shells, represent another class of nanoparticle structures suitable for drug delivery [85]. Nanocapsules have also been designed for efficient drug transport and controlled release [86]. Furthermore, nanobubble water has been applied in root canal therapy to improve smear layer removal and enhance the effectiveness of disinfectants in regenerative treatments [87].

### 3.2 Hydrogel restorative materials

Hydrogels can be classified into natural and synthetic types based on the origin of their polymer matrix. Natural hydrogels [88] are primarily derived from polysaccharides and fibrous proteins [89], [90], [91], [92], whereas synthetic hydrogels are formed via physical or chemical cross-linking of polymers, including polyacrylic acid and its derivatives [93], [94], [95], [96].

**Fig. 4:**
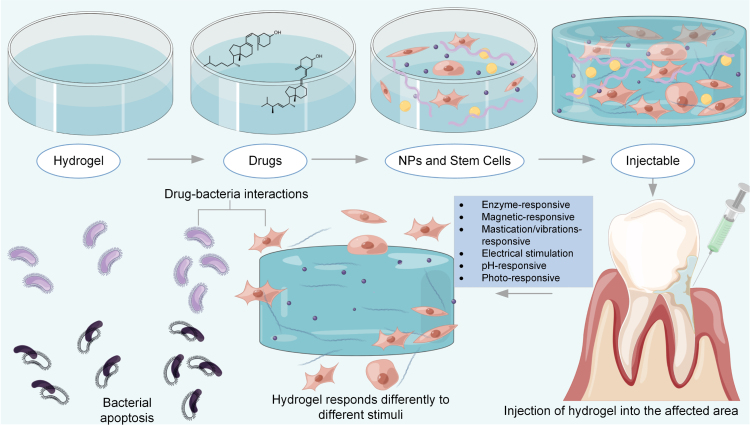
Process of hydrogel fabrication and the therapeutic process in the treatment of periodontitis.

On the basis of the mode of cross-linking, hydrogels can be divided into chemical and physical types [97]. Physical hydrogels are reversible and primarily stabilized through non-covalent interactions such as chain entanglement and hydrogen bonding. In contrast, chemical hydrogels consist of 3D polymer networks formed by strong covalent bonds, rendering them irreversible. Hydrogels can also be categorized according to polymerization method into homopolymerized, copolymerized, and interpenetrating polymerized types, or by electrical properties into nonionic, ionic (anionic and cationic), and amphiphilic hydrogels [98]. Additional classifications include biodegradability (biodegradable vs. non-biodegradable) and responsiveness to external stimuli (environmentally responsive vs. unresponsive). Fig. 4 illustrates the fabrication process of chemically cross-linked hydrogels and their mechanism of action in periodontitis.

#### 3.2.1 Hydrogels from natural materials

The development of efficient antimicrobial systems using nanotechnology has significantly advanced biomedical applications. Nanogels, which are soft polymer particles with internally cross-linked structures, behave similarly to hydrogels and can reversibly swell or shrink in response to solvents and external stimuli [99]. Fig. 4 schematically depicts the hydrogel fabrication process and its sterilization mechanism during drug delivery.

Since NACPs release drugs only upon degradation by specific enzymes, their efficacy can be enhanced by modifying the substrate with peptide sequences or optimizing external conditions to achieve precise release kinetics and cell adhesion properties [100]. Hydrogels can be designed to respond to various stimuli, such as chemical cross-linking for controlled drug release [101], temperature-dependent adhesion to target cells or tissues [102], pH-induced responses [103], [104], or dual-responsive bifunctionality [105], [106]. Slow-release strategies can further enhance the therapeutic efficacy of antibiotics [107].

Nanogels can act as carriers for essential oils, improving antimicrobial performance [108], and can be engineered for tissue protection and localized delivery of molecular or cellular therapeutics [109]. Natural material-based hydrogels are also employed in soft tissue engineering, promoting cell proliferation and migration in vitro [110] or reducing drug administration frequency in periodontitis treatment [111]. Hybridized hydrogels, combining natural polymers (e.g., gelatin, chitosan, hyaluronic acid), synthetic polymers (e.g., polyvinyl alcohol, polyurethanes), and inorganic substances (e.g., nanoparticles, hydroxyapatite), integrate biocompatibility and hydration with strong tissue adhesion and free radical scavenging, showing great potential for antimicrobial wound dressings.

Pulp-derived extracellular matrix hydrogels, fabricated from bovine molar extracts and tested in canine models, retain bioactive properties and demonstrate potential for pulp regeneration surgeries [112]. Natural hydrogels also serve as bioactive agents. For example, an injectable chitosan/hyaluronic acid/heparin hydrogel that promotes angiogenesis shows promise for bone regeneration and drug delivery applications [113]. Serum albumin microspheres doped with ZnO NACPs, incorporated into Carbopol 940VR hydrogels, exhibited superior therapeutic efficacy and gingival tissue repair compared with 2% minocycline ointment (PerioVR) [114]. Multifunctional hydrogel-based stem cell microhabitat engineering strategies have also been developed for treating inflammatory bone loss [115]. Nanohydroxyapatite particles can be used to fabricate composite scaffolds for promoting bone and soft tissue regeneration [116], while injectable smart hydrogels containing free or nano-encapsulated leflunomide (LEF) enhance cartilage repair, limit chondrocyte apoptosis, and provide synergistic targeting [117].

Additionally, silk threads can be incorporated into hydrogels, such as L-lysine-based nanogels grafted with triclosan-loaded nanogels, s which are prepared via enzymatic degradation of macromolecular gels [118].

#### 3.2.2 Synthetic hydrogels

Natural hydrogels are sometimes limited by poor mechanical strength, making them unsuitable for load-bearing applications. In contrast, synthetic hydrogels offer enhanced stability, and their porosity, degradation rate, drug release profiles, and mechanical properties can be tailored via chemical modification of specific structural units. Table 1 presents a comparison of commonly used hydrogel materials and key parameters.

**Table 1:** Comparison of common materials used to make hydrogels with some basic parameters.

Material/Gel	Agents/NPs	Drugs	CL	Res	Advantages	Disadvantages	APPL	TRL	Ref
α-poly-L-lysine	Sodium alginate	Interferon-β	Ionotropic	ENZ	Specific release Cells safety	Stability Biocompatibility	DDST	Low	[157]

CS	Polyethylene oxide	MTR	Chemical	GOX	MS Biocompatibility	Slow degradation rate	DDST	Medium	[137]

Tamarind seed xyloglucan	Methylcellulose	MTR	In situ forming	T	Injectability Hydrophobic	Poor adhesion	DDST	Medium	[158]

CMC	LDHs	AMX	Physical	pH	Cell safety	Stability Mechanical	DDST	Medium	[138]

Water-soluble CS	Alginate blended	Protein drug	Genipin	pH	Cells safety Amount released	Rapid degradation	DDST	Low	[155]

CHC, β-GP, Glycerol	N/A	Naringin	Chemical	pH/T	Release rate Gelation rate Injectable	Synthetically complex	DDST	Low	[159]

CS, β-GP, Gelatin	N/A	Aspirin, EPO	Chemical	T	Injectability Cells safety	Temperature-sensitive Limited load	DDST/GTR	Medium	[121]

MMP2, KMP2	N/A	MSC-EVs	Nanofiber	ENZ	Slow release Cells safety	Stability High cost	DDST/GTR	Low	[94]

CCA nanogel	N/A	PEOs	Physical	AFs	Encapsulated Antimicrobial	Stability Poor scalability	DDST	Low	[160]

Functional gels	Cyclodextrin host	N/A	In situ forming	pH	Antimicrobial Cells safety	Mechanical Unstable drug release	DDST	Low	[161]

Gelatin	N/A	AMP	Curing systems	pO	Cytocompatibility Antimicrobial	Light curing Variability	BAs	Medium	[162]

Poloxamer, Chitosan	N/A	Moxifloxacin	In situ forming	T	Adhesion Release time	Solvolysis	DDST	High	[163]

HA	N/A	PDA-HA, PDA	Michael addition	T	Mechanical strength Antimicrobial	Complex Preparation Color Interference	Wound Management	Medium	[164]

CS, HA	N/A	Hep	Sol–gel technique	pH/T	Injectable Cells safety	Unstable Gelation Poor Compatibility	DDST/GTR	Low	[165]

Carbopol 940VR, Zinc oxide	MI	Albumin microspheres	Physical	pH	Release time Antimicrobial	pH-Sensitivity Ion Instability	GTR	Medium	[92]

Gel-based stem niche	N/A	EMSCs	Chemical	T	Injectable Cells safety	Instability Degradation Risk	DDST/GTR	Low	[68]

Biomimetic polysaccharide	Hydroxyapatite	Composite scaffold	Physical	pH	Injectable Self-repairing	Brittleness Limited Self-Healing	TE/GTR	Low	[102]

HP, CS/β-GP gels	CHS, HA	LEF	Chemical	T	Injectable Antimicrobial	Thermal Instability Complex Formulation	GTR	Low	[166]

L-lysine based nanogel	Antibacterial silk	Triclosan	Degradation	ENZ	Cell safety Degradability	Enzyme-Dependence Immunogenicity Risk	DDST	Low	[29]

PEG-DA, DTT, FPM	N/A	SDF-1	Michael addition	Gingipain	Quantitative release Cells safety	Reaction Inhibition	DDST/GTR	Medium	[63]

GelMA, PEGDA	N/A	PDLSCs	Chemical	pO	Bioprinting Injectable	Photoinitiation Cytotoxicity	GTR	Medium	[51]

PGBSs, CTPC	N/A	MI, BSA, KSL	Michael addition	MMP-8	Cell safety	cost Dependence	DDST	Low	[167]

CS	PCL	TCS, FLB	Chemical	pH/T	Antimicrobial Cell safety	BurstRelease Acidification	DDST	Low	[168]

GPLD polypeptides	Fe^3+^	Cancer drug Metal NPs	ENZs Oxidized, Fe^3+^	pH	Antimicrobial Mechanical strength Degradability	Complex preparation Metal ion leakage risk	DDST	Low	[169]

HBDLDC	N/A	CIP, NB	Thiol-ene click	N/A	Biocompatibility Degradability	Lack of stimuli-response Limited functionality	TE	Low	[170]

HA-SH gel	Cyclic onitro benzyl	N/A	Photo	pO	Release time Degradable	UV-curing required Limited functionality	BAs/GTR	Low	[14]

GelMA Photoinitiator	N/A	MTR, CHX	Chemical	pO	Biocompatibility Cell safety	Initiator Toxicity Photo-Damage	DDST	Medium	[132]

GelMA, SilMA gels	N/A	GMSCs	Physical	pO	Injectability Biocompatibility	UV-Curing Required Altered Properties	GTR	Medium	[25]

Sodium alginate gel	N/A	Bi_12_O_17_Cl_2_ Cu_2_O NPs	Chemical	pO	Demand release Biocompatibility	Ion Instability Initiator Toxicity	DDST	Low	[27]

P(CBMA-co -DMAEMA)	N/A	AMPs	Physical	pH	Biocompatibility Stability	Limited Loading Unknown Fate	DDST	Medium	[171]

Material/Gel: Chitosan (CS), Carboxymethyl cellulose (CMC), Carboxymethyl-hexanoyl chitosan (CHC), Matrix metalloproteinase-2 (MMP2), Sensitive self-assembling peptide (KMP2), Chitosan-cinnamic acid (CCA), hyaluronic acid (HA), Hyaluronic/pluronic (HP), Polyethylene glycol diacrylate (PEG-DA), dithiothreitol (DTT), Functional peptide module (FPM), GelMA, Poly(ethylene glycol) dimethacrylate (PEGDA), Polyethylene glycol-based scaffolds (PGBSs), Cysteine-terminated peptide crosslinker (CTPC), L-Dopa conjugated (GPLD), Hyper-branched dendritic-linear-dendritic copolymers (HBDLDC), GelMA, Silk fibroin glycidyl methacrylate (SilMA), β-sodium glycerophosphate (β-GP), pH-responsive carboxybetaine methacrylate-dimethylaminoethyl methacrylate copolymer P(CBMA-co-DMAEMA).

Agents/NPs: Layered double hydroxides (LDHs), Minocycline (MI), Chondroitin sulfate (CHS), Poly-ϵ -caprolactone (PCL), Ciprofloxacin (CIP), Sodium salt (NB), Metronidazole (MTR), Chlorhexidine (CHX), Gingival tissue-derived MSCs (GMSCs).

Drugs: Metronidazole (MTR), Amoxicillin (AMX), Erythropoietin (EPO), Mesenchymal stem cell-derived extracellular vesicles (MSC-EVs), Peppermint Essential Oils (PEOs), Antimicrobial peptide (AMP), HA-based hydrogel (PDA-HA), Polydopamine (PDA), Heparin (Hep), Ectomesenchymal stem cells (EMSCs), Leflunomide (LEF), Stromal cell derived factor-1 (SDF-1), Periodontal ligament stem cells (PDLSCs), Bovine serum albumin (BSA), Antibacterial peptide (KSL), Triclosan (TCS), Flurbiprofen (FLB).

Res: Enzymes (ENZ), Glucose oxidase (GOX), Temperature (T), Aspergillus flavus (Afs), Photorespose (pO).

APPL: Drug Delivery Systems Technologies (DDST), Guided Tissue Regeneration (GTR), Bonding agents (Bas), Tissue Engineering (TE).

Periodontitis, a major oral disease leading to tooth loss, is characterized by chronic inflammation of the gingival tissue, formation of periodontal pockets, and destruction of the supporting bone and connective tissue. Bacterial colonization of subgingival plaque triggers immune responses, resulting in tissue damage. Existing topical delivery systems for periodontitis often exhibit suboptimal antimicrobial efficacy and inadequate promotion of periodontal regeneration. To address this, a protein-responsive smart hydrogel (PEGPD@SDF-1, polyethylene glycol-polydopamine@stromal cell-derived factor-1) has been developed for targeted drug delivery [119]. Stem cell-based therapies combined with injectable hydrogels are emerging as promising strategies for repairing alveolar bone defects in periodontitis [120], as shown in Fig. 4.

Biodegradable, stimuli-responsive hydrogels enable in situ adaptive degradation for chronic periodontitis and peri-implantitis, overcoming limitations of short retention times and low localized drug concentrations [121]. Dual-action nanogels have been designed to provide superior therapeutic efficacy compared with physical drug mixtures [122], [123], [124]. Similarly, hyperbranched dendrimer-linear-dendrimer (HBDLD) copolymer-based gels demonstrate promise as biocompatible, degradable dual-delivery systems.

The humid and dynamic oral environment poses challenges for localized treatment of mucosal diseases. Photocrosslinked hydrogel adhesives can create favorable microenvironments for tissue repair, potentially reducing healing time [125], [126]. For peri-implantitis, multifunctional, temperature-sensitive hydrogels based on chitosan (CS) and β-glycerophosphate (β-GP) undergo gelation at physiological temperatures. Drug loading and pH sensitivity can be enhanced by incorporating simvastatin into zeolite imidazolate framework-8 (ZIF${}_{8}$), forming Sim@ZIF${}_{8}$@PDA NACPs. Coating with polydopamine (PDA) improves photothermal efficiency and stability, and integration with CS/β-GP hydrogels helps alleviate infection and inflammation. Photocrosslinked gelatin-methacryloyl (GelMA) hydrogels are widely applied in regenerative engineering due to their excellent cell-tissue affinity and degradability in the presence of matrix metalloproteinases [127]. Hydrogels loaded with minocycline (MTR) and CHX, such as UV-crosslinked gGels (MTR@gGels) and CHX (CHX@gGels), may have potential applications in endodontics for root canal disinfection [128].

Injectable photocrosslinked porous GelMA/silk protein–glycidyl methacrylate (SilMA) hydrogels encapsulating gingival mesenchymal stem cells (GMSCs) represent a novel strategy for peri-implant epithelium integration [129]. To enhance biofilm disruption, targeting, and minimize enamel damage, an injectable sodium alginate hydrogel membrane doped with Bi_12_O_17_Cl${}_{2}$ and Cu${}_{2}$O NACPs has been developed [130]. This hybrid hydrogel layer exhibits antifouling properties, effectively resisting bacterial adhesion under physiological conditions [131].

### 3.3 Natural agents and extracts

**Fig. 5:**
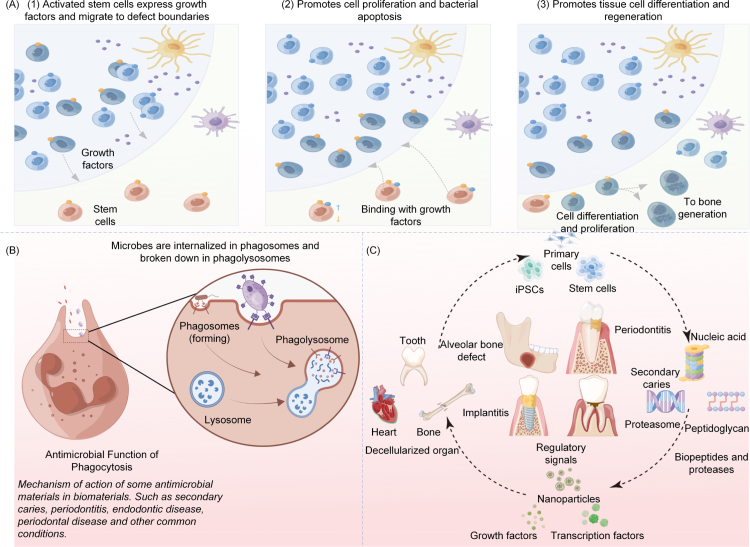
(A) Growth factors promote cell proliferation and differentiation. (B) Tissue sterilization and anti-inflammatory action mechanism. (C) Sources of biomaterial components.

Natural agents and extracts have diverse applications in restorative dentistry, including biological proteins with antimicrobial activity, particularly antimicrobial peptides (AMPs). Autologous tissues, such as bone and dental-derived growth factors, are also employed. When combined with suitable materials, these agents can offer innovative solutions to longstanding dental challenges. Two common modes of action for biologics in tissue engineering, namely cell proliferation and bactericidal activity, are illustrated in Fig. 5 (Panels A and B). Examples of widely studied natural agents and extracts, including AMPs and autologous bone, are presented in Fig. 5C.

#### 3.3.1 Antimicrobial peptides

AMPs are a promising class of antimicrobial agents due to their reduced susceptibility to bacterial resistance development [132]. This advantage arises from their distinct mechanism of action and pharmacodynamic properties, which differ substantially from traditional antibiotics. Consequently, AMPs are increasingly incorporated into dental restorative materials. Bisensitive antimicrobial peptides are particularly effective at selectively targeting bacterial biofilms, offering a potent strategy for preventing and treating dental caries [133]. For example, ϵ-polylysine exhibits antimicrobial activity against oral pathogens, including *Streptococcus pyogenes* and *Porphyromonas gingivalis*, significantly reducing viable cell counts [134].

GH_12_, a potential smart agent for caries prevention, specifically targets acidic microenvironments, enhancing its antimicrobial and antibiofilm activity in cariogenic conditions [135]. The presence of biofilms and the increasing prevalence of drug-resistant bacteria diminish the efficacy of conventional antibiotics. Immobilizing AMPs on implant surfaces offers a promising alternative for preventing implant-associated infections (IAIs). For instance, AMP-coated silicone catheters and titanium surgical implants exhibit pH-responsive antimicrobial activity [136], [137]. Tethering AMPs with functional linkers can further enhance their antimicrobial efficacy and biological activity, with effectiveness dependent on the target cell type [138]. This strategy includes extracellular matrix chimeric systems based on elastin-like recombinants [139].

AMP sequences typically possess numerous reactive groups (e.g., amines, carboxylic acids) on their side chains. Direct covalent coupling can result in uncontrolled grafting, random orientation, and non-uniform surface densities [140]. Rational design of AMPs, including modulation of their physicochemical properties, offers opportunities to optimize antimicrobial performance [141], [142]. Various surface modification methods, such as polymer brushes, enhance bacterial attachment prevention by immobilizing more peptides without contamination from polymeric PDMA fragments [143]. Filipin proteins are also widely employed in biotechnology as promising antimicrobial agents [144]. Dual-layer protection strategies, such as priming or coating dentin with amphiphilic and antimicrobial peptides (AAMPs), help resist recurrent caries around bonded restorations [145], [146]. Moreover, incorporation of the antimicrobial peptide lactobacillin into a single-bond universal adhesive has been shown to significantly inhibit the growth of *Streptococcus pyogenes* monospecific and salivary-derived multispecies biofilms [147]. However, few studies have explored peptidergic antimicrobial binder copolymer systems that primarily rely on nonspecific adsorption [148].

#### 3.3.2 Autologous bone

Bone tissue engineering holds significant potential for regenerating complex bone structures, from orthopedics to oral and maxillofacial applications. A critical property of biomaterials in this field is their ability to integrate seamlessly with the patient’s body. Among the various graft materials used in dental practice, autologous bone remains the gold standard [149]. Its primary limitations, however, include finite availability, inevitable resorption, and the potential for secondary defects at the donor site. Gingival mesenchymal stem cells isolated from discarded gingiva demonstrate robust in vitro growth and osteogenic differentiation, representing a promising approach for cost-effective and minimally invasive autologous bone regeneration [150].

Similarly, autogenous dental particle biomaterials derived from extracted human teeth exhibit strong antimicrobial properties, outperforming bioactive glass ionomers and calcium hydroxide cements [151], [152]. Recently extracted dental particles have been applied in the posterior canals of dogs, confirming that immediately transplanted autologous dentin particles serve as effective graft materials. These particles are rich in growth factors, promoting more predictable and favorable bone formation at the graft site [153], [154]. Traditional osteogenesis assessments often require invasive procedures; however, qualitative and quantitative evaluations of osseointegration and osteogenesis provide non-invasive alternatives [155]. Additionally, quantitative, efficacy-based strategies, such as periosteal substitution membranes, facilitate tissue regeneration across diverse surgical and regenerative medicine contexts [156].

### 3.4 Chemical and resin-based smart materials

Chemical and resin-based materials are widely employed for dental defect restoration due to their color similarity to natural tooth tissue and favorable biocompatibility. Composite resins are generally classified as universal or posterior-specific types, with wear resistance determined by filler content, particle size, matrix composition, and interfacial bonding strength. Matrix erosion typically precedes filler detachment, and surface roughening promotes plaque accumulation and secondary caries. Increasing filler content and reducing particle size can enhance mechanical performance [172], [173].

Various functional additives have been investigated to confer self-repair, antimicrobial, and remineralization properties, including polymerizable monomers [160], TU oligomers [174], bioactive glass adhesives [175], fluoride-releasing systems [176], and TCN-HNT nanotubes [177]. Direct-current regulation of bond strength has also been explored [178].

Resin reinforcement is commonly evaluated using bond strength, microleakage, and cervical margin integrity [179], [180], with outcomes influenced by parameters such as light-curing units, sandwich techniques, and interface design [181], [182], [183], [184], [185], [186]. Excessive polymerization shrinkage may induce debonding at the cavity base [187], whereas vertical printing combined with extended light-curing can mitigate this effect [188]. Post-curing times generally range from 20 to 60 min for optimal polymerization, with 30 min commonly applied for certain light-cured resins [188]. Material performance is also assessed via degree of conversion and polymerization shrinkage [189]. Fracture toughness of resin-bonded cement depends on storage time and medium [190], while self-healing dentine cement containing microcapsules significantly improves both toughness and repair efficiency [191].

Bulk-fill resins often exhibit high wear rates and low hardness, limiting their suitability for high-stress applications [192]. Sculptable fillers require longer curing times than flowable types [193], and low-viscosity bulk materials show lower microhardness than high-viscosity resins, necessitating overlay compensation [194]. Nanocrystalline- modified DCPA fillers support mineralization while maintaining mechanical strength [195]. Multi-band LED curing achieves superior strength-to-weight ratios compared to single-band curing [196], and high-intensity light curing further enhances radial tensile strength [197]. Curing 4 mm bulk specimens did not induce significant cytotoxicity [198]. Non-destructive, real-time bioluminescence detection has been proposed for quantitative evaluation of *Streptococcus mutans* biofilms on composite resins [199].

Advances in materials science, combined with increasing clinical demands, have enabled chemical and resin-based smart restorative materials to achieve multifunctionality, including antibacterial activity, remineralization, self-healing, and controlled release. Through the incorporation of functional molecules, ions, and nanostructures, these materials can be designed as single-stimulus-responsive or multi-stimulus-coupled-response types, facilitating clearer description of their mechanisms and enhancing clinical application potential.

#### 3.4.1 Single-stimulus responsive materials

**Fig. 6:**
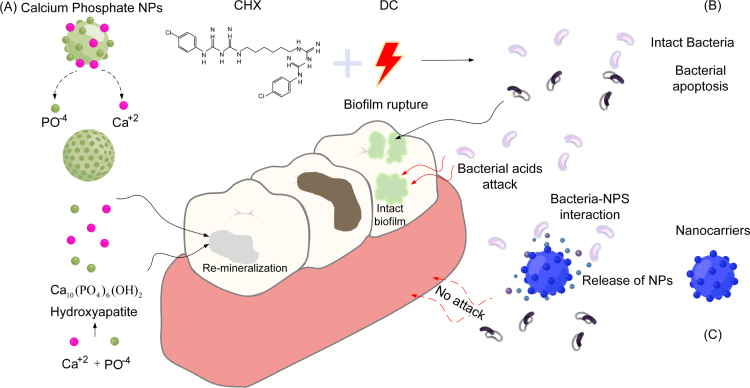
Mechanism of action of chemical agents. (A) NACPs support remineralization. (B) Weak current enhances the bactericidal effect of CHX. (C) NACPs release biocidal substances.

pH-responsive and localized antimicrobial release are critical for controlling mucositis and peri-implantitis, as they influence biofilm formation and microbial activity around oral implants. CHX, a widely used broad-spectrum biocide with low toxicity, is frequently incorporated into surface coatings and carrier systems to enhance the antimicrobial efficacy of restorative materials and implants. Despite its advantages, CHX has limitations: prolonged use can cause tooth discoloration (typically brown or yellow) and oral mucosal irritation [12], and evidence suggests long-term use may promote bacterial resistance, potentially diminishing clinical efficacy [13]. Therefore, careful management of CHX application is required to maximize antimicrobial effectiveness while minimizing side effects. Representative clinical and research applications include PIXIT implants with a polysiloxane oligomer coating containing 1% CHX gluconate [200]; combination of low-intensity direct current with CHX to inactivate *Streptococcus mutans* and *Staphylococcus aureus* biofilms [201]; and dentin pretreatment with 2% CHX to inhibit secondary caries and reduce marginal gaps (Fig. 6B illustrates direct current enhancing CHX’s anticaries effect) [202].

Smart and controlled release strategies have been developed using pH-responsive nanocarriers for CHX delivery [203]. CHX/ACP NACP embedded in resins simultaneously enhance remineralization and antimicrobial activity (Fig. 6A) [204]. Functionalization of CHX onto Fe${}_{3}$O${}_{4}$-NACP particles allows regulation of release kinetics via external magnetic fields [205]. CHX-loaded poly(ϵ -caprolactone) nanocapsules (nano-PCL), prepared via interfacial polymerization, enable targeted delivery along microdentin tubules to demineralized dentin and the resin–dentin interface [206]. CHX-methacrylated α-tricalcium phosphate composites serve as root canal sealants, improving physicochemical and antimicrobial properties while promoting periapical healing [207]. Additionally, CHX⋅HCl nanoemulsions enhance penetration and cleaning efficiency in root canal irrigation [208]. Injectable and UV-crosslinked hydrogels loaded with MTR and CHX, as well as photopolymerizable GelMA-modified CHX nanotubes, provide viable clinical infection-control strategies [209], [210]. Polymer-based antibiotic delivery systems and minocycline microspheres coated with calcium phosphate polymer layers offer sustained-release pathways for periodontitis treatment [211], [212].

Photoresponsive and photopolymerizable systems have also been applied in dental restorations. CHX-nanotube-modified injectable GelMA hydrogels enable light-triggered localized release and antibacterial effects [210]. Optimization of matrix monomers, photoinitiators, and filler compositions further improves the photopolymerization depth and curing efficiency of bulk-fill resins.

Electrical stimulation can synergistically enhance antimicrobial activity. Low-intensity direct current has been shown to remove oral biofilms in combination with CHX [201] and modulate bond strength at the resin–dentin interface [178]. Thermally or chemically triggered self-healing and microcapsule systems provide exogenous-assisted repair: microcapsules or microchannels embedded in the resin rupture upon crack propagation, releasing healing fluids that react with exposed powder to form insoluble products, sealing the crack and extending material lifespan [168], [213], [214], [215], [216], [217], [218]. Healing efficiency depends on microcapsule size, concentration, silane treatment, and healing temperature. Representative systems include PUF-shell microcapsule self-healing adhesives with TEGDMA as the healing fluid [219] and self-healing resins based on polymerizable dental monomers [160]. Incorporating thiourea (TU) oligomers reduces internal stress and enhances fracture toughness without compromising conversion rates [174]. Bioactive Glass resin adhesives and fluoride-releasing smart materials exhibit “smart reactivity” under acidic conditions [175], [176].

Functional monomers and coatings activated by single stimuli have been employed to enhance targeted antimicrobial activity and reduce microleakage. Examples include antimicrobial methacrylate/ methacrylamide monomers, fluoride-releasing composites, and molecules such as DMAEM (pH-responsive) and QPS (enhanced activity at low pH) [220], [221], [222], [223], [224]. I-HAp (Sr/Zn/Mg-doped hydroxyapatite)-SiNT composite coatings and superhydrophobic protective sprays (ZFPs) improve biocompatibility and corrosion resistance but provide limited infection protection [225], [226]. Graphene oxide coatings enhance implant-soft tissue integration and serve as barrier and mechanical reinforcement materials [159], [227], [228].

#### 3.4.2 Multi-stimulus coupled response materials

In antibacterial–remineralization coupled systems, integrating antimicrobial functionality with remineralization agents is a major research focus. CHX/ACP (NACP) embedded in resins provides both antimicrobial activity and promotes remineralization [204]. Phosphate-Zn-Al layered double hydroxides (LDH) allow sequential phosphate release, contributing to dentin remineralization [221]. Fluoride-releasing antimicrobial composites provide additional synergistic pathways for caries prevention [220].

Coupling antimicrobial activity with self-healing or interface stability has been demonstrated using specific monomers or fillers. These materials improve bond durability by inhibiting matrix metalloproteinases while providing antimicrobial efficacy. For example, MDPB functions as a potent antimicrobial monomer and suppresses MMP activity at the resin–dentin interface, enhancing the durability of self-etch adhesives [163], [169]. DMAHDM exhibits dose-dependent antibacterial effects, and its integration with self-healing microcapsule systems achieves both antimicrobial efficacy and increased toughness [191], [229].

Functionalized nano- and bioinspired layered structures offer additional multi-stimulus coupling strategies. Epidermis-like layered designs, combined with the mechanical and barrier properties of GO, support self-healing in hard materials [227]. GO nanosheets enhance mechanical performance while fulfilling biomedical requirements in oral and orthopedic applications [228]. Alginate microspheres serve as carriers for cells and drugs in bioreactors, supporting osteogenic differentiation and promoting mineralized matrix deposition, enabling cell-smart systems with enhanced osteogenic potential [230]. Poly(ϵ -caprolactone) (PCL) 3D scaffolds fabricated via fused electrowriting guide hPDLSC differentiation and modulate macrophage polarization, achieving simultaneous immune regulation and tissue regeneration [231].

Coupled effects of external fields and processing parameters further extend material functionality. Multi-band LED light sources achieve higher curing intensity ratios in composite resins compared to single-band sources [196], and high-intensity light curing maximizes radial tensile strength [197]. Direct current electric fields can modulate bond strength [178], whereas magnetic fields can regulate drug release from Fe${}_{3}$O${}_{4}$ carriers [205]. Interactions between external stimuli and material interfaces provide technical strategies for controlled release with tunable adhesion in clinical applications.

### 3.5 Intelligent carrier materials

Smart carrier materials have significant potential in dental restorations, particularly for developing antimicrobial restorative materials. For example, pH-responsive Zn-releasing glass particles have been used to create smart antimicrobial materials that exhibit pronounced antimicrobial effects. The “smart” behavior of these materials arises from their ability to dynamically release active ions in response to environmental stimuli, rather than from inherent smart properties of the glass itself. Ultrasonic vibration technology can improve the adhesive strength and durability of glass fiber posts, thereby enhancing restoration performance.

Two-dimensional metal–organic framework materials demonstrate superior antimicrobial activity through the release of reactive oxygen species and ions. These metal–organic frameworks exhibit smart behavior by actively releasing antimicrobial species when triggered by environmental cues. When combined with aPDT, these materials provide a promising strategy for periodontitis treatment. Additionally, glass ionomer materials have shown potential in preventing enamel and dentin demineralization. However, combining these materials with silver diamine fluoride (SDF) may reduce their bond strength, highlighting the need for optimization to ensure compatibility with other restorative materials while maximizing therapeutic efficacy.

#### 3.5.1 Metal materials

Peri-implantitis, a severe oral disease, has created demand for improved implantable dental biomaterials. This condition is characterized by biofilm-induced inflammation, leading to rapid bone loss around implants. Unlike natural teeth, the lack of a robust soft tissue seal (analogous to the periodontal ligament) around implant necks facilitates bacterial infiltration, making inflammation more aggressive and harder to control than periodontitis.

Nanometals can integrate effectively with supporting bone and resist bacterial colonization [232]. Nanostructured titanium, in particular, has emerged as an ideal material. Extensive literature demonstrates its in vitro efficacy against aerobically cultured bacteria [233]. Hydrothermal etching has been employed to design sharp, needle-like nanostructures on commercially pure titanium surfaces, mimicking the bactericidal effect of insect wings. These surface modifications also enhance the efficacy of azithromycin against anaerobic dental pathogens compared to unmodified titanium [234]. Nevertheless, further studies are needed to understand the performance of nanostructured biomaterials in anaerobic environments like the oral cavity [165].

Systemic or localized blockade of  β,  , or  α around titanium implants has been proposed as a therapeutic approach for peri-implant bone loss [235]. Titanium–titanium implant abutment interfaces exhibit greater resistance to damage than titanium–zirconia interfaces [236], but long-term mechanical adaptation depends critically on surface morphology. Stress shielding due to elasticity mismatch between bone and implant remains a major concern. Various surface treatments, including laser micromachining, have been developed to modify titanium implant surfaces in a non-contact, contamination-free, and flexible manner [237], [238]. The heterogeneous structures of 2D metal–organic frameworks have also been utilized to achieve antimicrobial properties through reactive oxygen species and ion release; when combined with aPDT, these materials are applicable for periodontitis treatment [239].

#### 3.5.2 Glass materials

On-demand release of antimicrobial components represents a promising design for smart restorative materials [240]. For instance, pH-responsive Zn${}^{2+}$-releasing glass particles with 42.7 mol% Zn have been developed for antimicrobial restorations. Sonic vibration techniques can improve the bond strength and durability of fiberglass posts by enhancing adhesive penetration into dentin and the flow of bonding agents [241].

Glass ionomers are effective in inhibiting enamel and dentin demineralization, as demonstrated by in situ evaluations [242]. However, SDF can negatively affect the bond strength of glass ionomer cement (GIC), particularly when applied to sound dentin before restoration [243]. In Class II cavities, bulk-filled and conventional composite resins outperform high-adhesion reinforced GICs in clinical performance [244], [245], although GICs still fall short of natural enamel in apparent mineral density [246]. VLC bioactive glass ionomers and calcium hydroxide cements, standard pulp-capping materials, also exhibit antimicrobial properties [247].

### 3.6 Ceramic and ceramic-based smart repair materials

Ceramic materials have shown considerable potential in tissue engineering and oral restoration due to their excellent biocompatibility, mechanical properties, and design versatility. Unlike conventional inert ceramics, recent studies have focused on introducing smart functionalities through functionalization and composite strategies, enabling these materials to provide both structural support and biological activity during tissue repair.

Metal–ceramic composite systems improve osteointegration while maintaining mechanical strength. Optimized titanium–hydroxyapatite and titanium–wollastonite composites exhibit superior physical and chemical properties, along with excellent biocompatibility with human bone progenitor cells, making them promising candidates for future bone repair implants [248]. In biodegradable metals, magnesium-based alloys treated with phosphorus ion implantation demonstrate enhanced electrochemical stability and corrosion resistance, while promoting favorable interactions with host tissues [249], [250]. These surface engineering approaches offer valuable guidance for designing ceramic/metal composites that integrate mechanical performance with biological functionality.

In 3D scaffold fabrication, techniques such as surface roughening, chemical functionalization, and nanoparticle coating significantly enhance cell adhesion and osteogenic activity in ceramic-based scaffolds [251], [252]. Scaffolds composed of carbohydrate–ceramic composites not only improve physicochemical properties but also expand the potential applications of advanced biomanufacturing. Furthermore, multifunctional ceramic-based nanocomposite scaffolds combine biocompatibility with antibacterial and antitumor activity. Incorporating graphene oxide, nano-hydroxyapatite, or polysaccharide-based biopolymers into the ceramic matrix enhances structural stability and functional diversity, facilitating the repair of complex bone defects [161], [253].

In summary, ceramic and ceramic-based smart repair materials are evolving from single-function structural supports to multifunctional, controllable, and intelligent composite systems. This trend broadens the material options for dental repair and extends the prospects for bone and soft tissue regeneration.

## 4 Applications of smart dental materials

Intelligent dental restorative materials can be broadly classified based on functional properties into intelligent drug delivery carriers, antimicrobial materials, regenerative materials, tissue scaffolds, and others. Depending on the specific restorative requirements and local environments, different material functionalities can be selected. These materials often exhibit multifunctionality; for instance, antimicrobial treatments developed for periodontitis may also be suitable for extra-oral wound dressings, while tissue engineering therapies for pulp regeneration could be adapted to repair alveolar bone defects. This subsection reviews the applications of intelligent dental restorative materials, summarizes their background, explains their mechanisms of action, and highlights potential future directions.

### 4.1 Drug delivery vehicles

Recently, topical drug delivery systems have been developed for a variety of oral diseases. These systems can be classified into biosoft tissue delivery and biosolid tissue delivery. Drug carriers must deliver therapeutics to specific sites, prolong retention, and reduce the required dose to minimize adverse effects. A key challenge is achieving targeted and precise delivery to narrow lesions, such as periodontal pockets or root canals within deep dentin tubules. This subsection discusses the application of different drug delivery systems in both hard and soft oral tissues.

#### 4.1.1 Bio-soft tissue

**Fig. 7:**
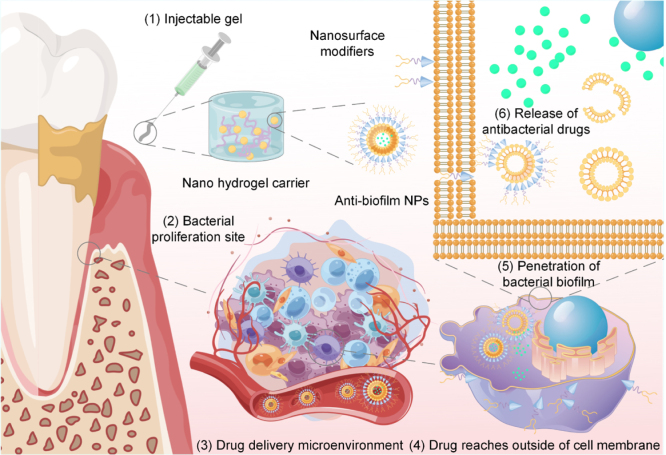
Drug delivery process and mechanism of action.

Various strategies have been proposed for drug delivery to biological soft tissues. The process and mechanisms of drug delivery, illustrated here using periodontitis as an example, are shown in Fig. 7. Biocompatible and degradable dual-delivery gel systems based on HBDLD, which degrade into non-toxic components, show considerable promise as wound dressing materials [254]. Polymers have also been explored as carriers for delivering antibiotics in the treatment of recurrent periodontitis [255]. Additionally, pH-sensitive carboxymethyl cellulose-based bio-nanocomposite hydrogel beads have been employed for oral delivery of amoxicillin (AMX) and controlled protein drug delivery [256].

Nanoparticle-stabilized liposomes combined with hydrogel technology provide more effective and sustained topical drug delivery [257]. Red blood cell membrane-coated nanogel (RBC nanogel) systems significantly inhibit bacterial growth compared with free antibiotics and non-responsive nanogels [258]. For soft tissue applications, injectable CHX-loaded nanotube-modified GelMA hydrogels have been developed, serving as efficient drug delivery systems for dental infection control [259]. Other strategies, including bionic hydrogels, pH-responsive hydrogels, slow-release systems, and sustained-release hydrogels, are under investigation as effective drug carriers for soft tissues [162], [260].

Microenvironment-activated PEG nanoplatforms facilitate targeted drug delivery into oral biofilms [261]. Antibiotic resistance and biofilm formation have been addressed through active antibiotic nanocarriers with protease-functionalized surfaces [262]. Topical delivery of nanohydroxyapatite as a reinforcing filler, combined with silver- metronidazole as a periodontal pocket disinfectant, demonstrates broad-spectrum antimicrobial activity with low systemic toxicity [263]. Electrospun pharmaceutical nanofibers enriched with antimicrobial hydroxyapatite layers can be fabricated for dental applications [264]. Moreover, monolithic self-assembled nucleoside NACPs are readily available, robust, biocompatible, and exhibit low toxicity [265].

#### 4.1.2 Biological hard tissue

Drug delivery applications in biological hard tissues primarily target surface enhancement and debridement of implants and autologous bone. Preventing implant-associated infections early is critical for the long-term success of dental and orthopedic implants. Traditional antibiotics are increasingly ineffective due to the emergence of multi-drug-resistant bacteria. Local delivery of antimicrobial agents provides a more effective strategy. AMP-eluting coatings on implant surfaces represent a promising alternative. For example, anodized TiO${}_{2}$ nanotubes have been utilized to deliver candidate AMPs on titanium surfaces, such as in modular osteochondral membranes, which function as adhesive gingival grafts and guided bone regeneration membranes at the soft–hard tissue interface while preventing oral infections. Dendritic polymers have also been employed for biomimetic remineralization of enamel and dentin, and pH- and thermosensitive hydrogels have been shown to promote angiogenesis.

Dental caries, a preventable infectious disease associated with biofilms, arises from interactions between oral bacteria and dietary sugars. Cariogenic bacteria, primarily *Streptococcus mutans* and lactobacilli, metabolize sugars to produce acids, locally lowering pH at the tooth–biofilm interface and dissolving hydroxyapatite crystals in enamel and dentin, a process known as demineralization. Given the acidic microenvironment of cariogenic biofilms, pH-sensitive drug delivery systems have emerged as innovative preventive materials [61]. Stimuli-responsive multidrug delivery systems, such as PMs@NaF-SAP, have been employed for caries treatment [7]. NPC-mediated drug delivery can be optimized by adjusting diblock properties, as exemplified by drug-carrying nanocapsules [53], [86]. Hydrogel NACPs with enzymatically degradable cross-linkers can be tailored to achieve controlled release kinetics and appropriate cell adhesion [100].

Beyond conventional drug delivery, smart tracking delivery systems offer a solution for precise targeting. Micro- and nano-motors can autonomously deliver antibiotic payloads to specific sites. Silica-based microrobots, catalytically powered by urease and incorporating antimicrobial peptides, combine navigation, catalytic conversion, and bactericidal activity to deliver antibiotics efficiently to infection sites, addressing drug-resistant pathogens [267]. Similarly, drug-loaded biodegradable micromotors enable autonomous directional motion and orientation control based on hydrogen peroxide gradients [268].

### 4.2 Tissue engineering scaffolds

**Fig. 8:**
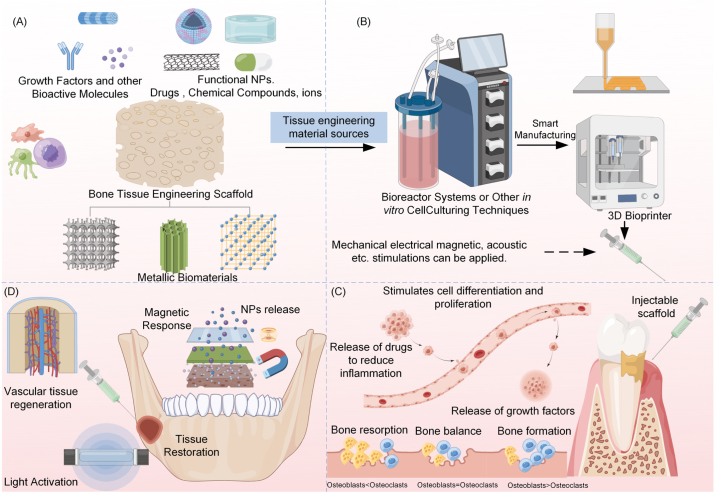
Tissue engineering materials originating from the mechanism of action. (A) Material source of the scaffold. (B) Fabrication process of the scaffold. (C) Substances in the scaffold promote bone repair. (D) Magnetic scaffolds to repair alveolar bone damage [266].

Bone repair and regeneration are governed by a combination of physiological cues, including biochemical, electrical, and mechanical factors, which act synergistically to achieve functional recovery, as illustrated in Fig. 8. Various materials have been explored as bioactive scaffolds (Fig. 8A) to locally deliver these cues at the injury site. For instance, piezoelectric materials can be fabricated into flexible 3D fibrous scaffolds that stimulate human mesenchymal stem cell differentiation and promote extracellular matrix formation under physiological loading conditions [269], [270] (Fig. 8B–C). Superparamagnetic scaffolds have also been developed [271], [272], exhibiting enhanced cell proliferation compared to non-magnetic scaffolds due to material biocompatibility and magnetization induced by an external static magnetic field (Fig. 8D). Additionally, 3D fused electrowritten scaffolds, such as poly(ɛ-caprolactone) constructs with tissue-specific properties, are available.

Injectable scaffolds are particularly attractive for dental and craniofacial tissue regeneration, offering versatile strategies for future development [273], [274] (Fig. 8C–D). To better mimic regenerated pulp tissue for endodontic therapy, hierarchical growth factor-loaded nanofibrous microsphere scaffolds have been designed [45]. These scaffolds protect growth factors from denaturation and degradation while enabling controlled release. Their fiber morphology elicits unique cellular responses [46]. Collagen scaffolds combined with MSCs from periodontally damaged gingiva provide a cost-effective and minimally invasive strategy for autologous bone regeneration [150]. Bionic polysaccharide hydrogels offer multifunctionality, including injectability, self-repair promotion, degradability, and a porous structure, supporting both bone and soft tissue regeneration [116]. Additionally, biodegradable stimuli-responsive hydrogels can be administered on demand [121].

### 4.3 Self-healing materials

Secondary caries at restoration margins is the leading cause of restoration failure, primarily due to microleakage at the restoration–tooth interface, which permits bacterial and fluid infiltration. Polymerization shrinkage of resins can create micro-gaps and cracks that provide niches for bacterial colonization and acid production, leading to recurrent decay. Self-healing materials are primarily applied in resin restorations, which are susceptible to fatigue-induced cracks that can trigger oral diseases, including caries.

**Fig. 9:**
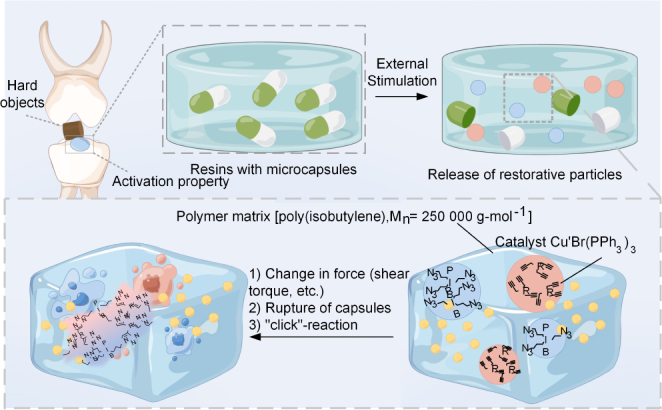
Repair mechanism of self-repairing capsules when encountering external stimuli from hard substances [158].

NACP-based systems and new strategies integrating self-healing microcapsules, DMAHDM, and NACP are being explored for dental adhesives, bonding agents, sealants, and composites. These approaches aim to address fractures and secondary caries [57], [82], [275], as illustrated in Fig. 9. Key components such as TEGDMA [56], [219], DHEPT [213], and PUF [215] are critical in composite resin repair.

The fracture resistance and self-healing capacity of self-healing dentine cement improve with increased microcapsule concentration [191], [214]. These properties are further influenced by microcapsule size, activator and initiator content, and healing temperature [218]. Microcracks formed during or after curing are sealed, extending composite service life [168]. Heat treatment can restore up to 85% of the original fracture toughness [217].

### 4.4 Antimicrobial materials

Natural antimicrobial materials are biocompatible, environmentally friendly, and less toxic, but face limitations such as limited availability, high extraction costs, poor stability, narrow antibacterial spectrum, low efficiency, and weak antimicrobial effects. Organic antimicrobial materials provide diverse options and effective antimicrobial properties but suffer from toxicity, poor heat resistance, easy decomposition, and volatility, raising concerns about long-term safety and potential drug resistance.

**Fig. 10:**
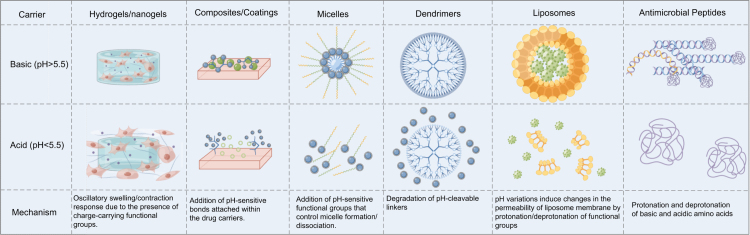
Configuration and release forms of pH-responsive vectors for antimicrobial therapies.

Inorganic antimicrobial materials, by contrast, exhibit broad spectrum activity, long lasting effects, and high temperature resistance, and do not readily induce bacterial resistance. Their drawbacks include complex production, high cost, poor stability, and limited duration of activity. Fig. 10 illustrates pH-responsive antimicrobial agents applied to materials.

#### 4.4.1 Surface modification and implant-related antimicrobial materials

Nanotechnology has greatly advanced the development of highly efficient antimicrobial systems, profoundly impacting the biomedical field [99]. Bioadhesive films with controllable and reversible underwater adhesion are widely applied in biosensing, drug delivery, and tissue regeneration. These films function as gingival grafts and guided bone regeneration membranes at the interface between hard and soft tissues, helping prevent oral infections [54]. For example, biodegradable, stimulus-responsive hydrogels have been synthesized to enable in situ adaptive degradation during peri-implantitis treatment [121]. Additionally, biocompatible materials with intrinsic anti-fouling properties, such as filaggrin protein, have emerged as promising candidates in biotechnology [144].

Local delivery of antimicrobial agents remains the most effective strategy to prevent bacterial infections. Accordingly, implant surface coatings or bioadhesives releasing AMPs exhibit considerable potential [110], [166]. Immobilizing AMPs on implant surfaces effectively prevents implant-associated infections [110], [139] and may modulate oral microbiota composition [200]. Surface design strategies that inhibit bacterial colonization via physical microstructures offer a novel approach for antimicrobial material development [143], [170], [276]. Importantly, the antimicrobial properties of restorative materials should be tailored to their specific microenvironments for optimal efficacy [186].

#### 4.4.2 Device-related and operational antimicrobial strategies

Device-related strategies include surgical instruments and interventional materials used during restorative procedures. Bacterial infections are the most common type of healthcare-associated infection. Silver and gold nanoparticles (NPs) have been widely incorporated into antimicrobial matrices [68], although challenges such as NP leaching, poor dispersion, and inconsistent reproducibility remain. To address these limitations, a smart antimicrobial hybrid matrix coating incorporating lysozyme-templated gold NPs into a polyethylene glycol/polybutylene terephthalate amphiphilic polymer matrix has been developed [71]. This coating demonstrates excellent biocompatibility and can be extended to other medical devices without compromising functionality. Furthermore, devices with sharp nanostructured surfaces can kill bacteria on contact, reducing reliance on antibiotics and minimizing the risk of secondary revision surgeries [48].

For restorative materials, microleakage of composite resin adhesives is a major contributor to sealant failure. Incorporating pH-responsive antimicrobial agent DMAEM into resin-based sealants [223] or adding antimicrobial peptide lactobacillus avidin to single-bond universal adhesives [147] significantly inhibits *Streptococcus pyogenes* and saliva-derived biofilms without compromising bonding performance. Nanotube-based adhesives (TCN-HNT) provide sustained antimicrobial activity while promoting mineral deposition [177]. Hybrid systems combining antimicrobial peptides with hydrophilic adhesives enhance bio-functionality at critical bonding/dentin interfaces [277]. Peptide-coupled dentin adhesives are considered a promising strategy to prevent secondary caries and improve the longevity of composite restorations [148].

For dentin pretreatment, AAMPs can reinforce fragile bonding interfaces [145]. An antimicrobial polyacid system has been achieved via one-step photoreduction of AgNPs in the polyacrylic acid solution of traditional GIC [73]. Self-healing adhesives containing DMAHDM and NACP repair microcracks, inhibit bacterial growth, provide ions for remineralization, and extend restoration lifespan [57], [82], [275]. The release of Ca and P ions, which prevent caries, is strongly influenced by NACP filler content and solution pH, highlighting its functionality as a “smart” material [58].

#### 4.4.3 Antimicrobial systems for restoration and tissue regeneration

Damage to restoration margins by cariogenic bacteria is a primary cause of restoration failure due to recurrent caries. Incorporating antimicrobial agents into adhesives reduces bacterial colonization at the interface, decreases recurrence, inhibits interface degradation, and extends restoration lifespan, while minimizing systemic exposure [131]. Direct incorporation of antimicrobial compounds into restorative materials faces limitations, including short release durations and potential compromise of material integrity. Embedding antimicrobial drugs into MSNs with high loading capacity and mechanical strength provides a foundation for intelligent antimicrobial restorative materials [80], [240].

Catalytic nanomaterials, such as NACPs, disrupt dental plaque biofilms [70], while nanoenzymes exhibit strong catalytic activity under acidic conditions [135]. pH-responsive drug delivery systems have emerged as promising tools for caries prevention [60], [61]. Additional advances include composite resins resistant to biofilm formation and protein adsorption, as well as fluoride-containing antimicrobial resins [220], [222]. Stimulus-responsive multi-drug delivery systems (PMs@NaF-SAP) offer new strategies for caries treatment [7]. Post-restoration, adhesives incorporating microcapsules, DMAHDM, and NACP enable self-healing, antibacterial, and remineralization functions [275]. Minimal CHX pretreatment of dentin reduces enamel marginal gaps [202], and combining CHX with degree of conversion treatment further enhances biofilm eradication [201], [208]. Antimicrobial peptides such as AAMP maintain bonding interface stability, with effects comparable to dual-sensitive antimicrobial peptides [133], [146].

Traditional surgical procedures and antibiotic therapy often have limited efficacy in chronic periodontitis, frequently resulting in tissue destruction, tooth loss, and systemic complications. aPDT demonstrates rapid antibacterial, anti-inflammatory, and vasculogenic effects [239], but is limited by shallow tissue penetration, insufficient oxygen supply, and incomplete inflammation clearance. Near-infrared (NIR) light strategies have been proposed to simultaneously generate O${}_{2}$ and CO [74]. Self-oxygenating nanocomposites combining red and infrared light stimulation, encapsulating amphiphilic silanes with Fe${}_{3}$O${}_{4}$ nanoparticles, e6 chloride, and coumarin 6, have also been developed [75]. Other NIR-triggered platforms use mesoporous silica-coated gold nanorods loaded with nitroso-N-acetylcysteine and indocyanine green [79]. Nanogels have potential for enhancing periodontitis treatment [122]. Research increasingly focuses on on-demand and sustained-release drug delivery systems, including ginkgo protein-responsive hydrogels [119] and in situ gel formulations [111]. Electrospun drug-loaded nanofibers combined with antibacterial hydroxyapatite layers are explored for periodontal defect repair [72], and glucose-sensitive antimicrobial hydrogels have been developed for diabetic patients [8].

Root canal systems present challenges as bacteria form resilient biofilms on dentinal walls and within complex anatomical structures. Methacrylate-based root canal sealers containing CHX and α-tricalcium phosphate exhibit antimicrobial activity and promote apical healing [207]. Photocrosslinked GelMA hydrogels are widely studied due to their high cell/tissue affinity and degradability by matrix metalloproteinases [127]. Other irrigants include red blood cell membrane-coated nanogels, MTR and CHX-loaded hydrogels [128], injectable photopolymerized hydrogels [10], and enzyme-crosslinked hydrogels loaded with metal nanoparticles [123]. Novel pH-activated nanoparticles have demonstrated potential for periodontal and root canal applications [51].

The rise of antibiotic-resistant bacteria poses a global threat. Conventional antibiotic treatments are limited by inadequate delivery, off-target effects, and promotion of resistance. Micro- and nanomachines capable of autonomous navigation and targeted antibiotic delivery have been developed to address these challenges. These systems integrate navigation, catalytic conversion, and bactericidal functions to deliver antimicrobial agents directly to infected sites [267]. degree of conversion treatments inhibit microbial biofilms and enhance antimicrobial drug efficacy through electrolytic and bioelectric mechanisms, including increased oxidative stress and improved antibiotic transport, particularly under low-intensity conditions [278], [279]. When combined with low-dose antibiotics, electric fields further improve biofilm treatment efficacy, a mechanism known as the “bioelectric effect” (BE) [280].

## 5 Discussions and future trends

Smart dental restorative materials are evolving from traditional passive fillers into multifunctional, intelligent systems that integrate self-healing, antimicrobial, remineralization, and regenerative capabilities. By incorporating nanotechnology, advanced hydrogels, and microencapsulation strategies, these materials not only restore function but also actively interact with the oral environment through controlled, localized, and responsive mechanisms. Emerging technologies such as 3D bioprinting and AI-driven material design are further accelerating innovation, enabling personalized and precision-oriented solutions. This transformation marks a critical step toward next-generation dental restoratives with enhanced clinical efficacy, long-term stability, and broader application prospects.

### 5.1 Development trends and application prospects of smart dental restoration materials

Dental restorative materials are transitioning from passive restorations to intelligent systems capable of responding to diverse diseases and environmental stimuli [164], [167], [281]. These smart materials exhibit advanced therapeutic functionalities, which can be broadly classified into four response mechanisms. First, stimulus-triggered release involves the controlled release of therapeutic agents in response to specific cues, such as pH-responsive nanoparticles or NACP (nanoparticles of amorphous calcium phosphate)-incorporated resins, which release bioactive ions under acidic conditions to inhibit bacterial biofilm formation and promote remineralization. The effectiveness of such systems is typically evaluated by drug release kinetics and biofilm inhibition rates. Second, spatially targeted delivery employs magnetically or electromagnetically guided nanocarriers, including magnetically controlled nanobots, to localize therapeutics at specific sites (e.g., dentinal tubules), with measurable outcomes such as targeting precision and localized therapeutic efficacy. Third, temporal control is achieved through stimuli-responsive hydrogels, such as GelMA, engineered to release drugs at specific time points following light, heat, or electrical stimulation. Relevant parameters for assessment include release lag time, duration of therapeutic action, and overall release kinetics. Fourth, efficacy-modulated response refers to multifunctional restorative materials, such as composite resins incorporating silver nanoparticles and ACP fillers, which simultaneously exert antibacterial effects and promote mineral deposition. Their performance is quantified by antimicrobial activity, calcium/phosphate ion release, and improvements in mineral content or mechanical hardness of treated tissues. Collectively, these intelligent functionalities represent a promising direction for next-generation dental restorative materials with precise, targeted responses.

Furthermore, biomaterials are increasingly designed to integrate multiple functions, enabling responses to diverse oral stimuli and producing synergistic effects [201], [208], [239]. For instance, smart materials can promote pulp tissue regeneration and vascularization [78], [87], [282], enhance remineralization to prevent caries [85], [204], [221], [275], and provide anti-inflammatory or analgesic effects for periodontitis [283]. This subsection highlights the limitations of current smart restorative materials, summarizes their research status, and outlines future development trends.

### 5.2 High-performance smart materials and advanced manufacturing technologies

Current trends in nano-repair materials involve modifying hydrogels, binders, and composite resins through the incorporation of other functional materials. For instance, carbon- and metal-based nanomaterials, due to their electromagnetic properties, can be integrated into hydrogel systems to achieve various stimulus-responsive behaviors. These properties are essential for applications in bio-tissue engineering, including on-demand, spatially controlled, and temporally regulated drug delivery [66], [147], [271]. Conjugated carbon-based nanomaterials, such as graphene and carbon nanotubes, possess electrical, mechanical, and optical properties that enhance GelMA networks, enabling hydrogels to respond dynamically to environmental cues. Applications include electroactive tissue engineering and artificial 3D cellular microenvironment scaffolds.

Protein corona formation on nanomaterial surfaces provides a crucial foundation for cell attachment. Recently, inorganic nanomaterials have been incorporated into hydrogels to enhance tissue engineering and drug delivery. Their chemical interactions impart diverse interfacial functionalities, improving the mechanical performance of hydrogels. Furthermore, the degradation behavior of nano-engineered hydrogels can be tailored for applications in both hard and soft tissue repair.

Nanobiomaterials based on metals and metal oxides, such as gold, iron, and silver, are widely utilized in drug delivery, conductive scaffolds, bioelectronics, and bioimaging. Gold provides conductivity, iron offers magnetic responsiveness, and silver imparts antimicrobial properties. These nanobiomaterials respond to both internal and external stimuli, making them suitable for cell encapsulation and selective release of genes, drugs, or proteins. Controlling the timing and localization of release enables intelligent drug delivery systems, particularly in dental restorations, adapting to diverse clinical environments.

The use of nano-fillers or blended nano-fillers in composite resins has been shown to inhibit plaque biofilm formation and balance demineralization and remineralization. Challenges remain, including: (1) Nanofiller agglomeration and the trade-off between antimicrobial efficacy and mechanical strength; (2) Diminishing long-term antimicrobial performance; (3) Cytotoxicity of nanofillers and potential NACP leakage, which require further safety evaluation.

Hydrogel restorative materials face several limitations. First, achieving high biocompatibility often compromises mechanical strength, which is critical for tissue engineering scaffolds. Although mechanical reinforcement strategies exist, the toxicity and degradation profiles of additives may restrict their applications. In load-bearing areas, metal fixation may still be necessary, conflicting with the minimally invasive advantage of injectable hydrogels. Second, uncontrolled or imprecise release of loaded substances can occur, such as sudden hydrogel degradation releasing bioactive factors prematurely.

Recent smart hydrogel systems allow controlled delivery in response to external stimuli. Beyond traditional triggers like pH, temperature, and light, hydrogels responsive to biochemical signals, including enzymes, antigens, and ligands, have been explored [281]. However, increasing functional complexity elevates cost and demands careful consideration of production, storage, and biosafety [164]. Standardized bone disease models for validation are lacking, and many hydrogels share similar compositions, gelation mechanisms, and bioactive functions.

Despite these challenges, hydrogels hold vast potential in smart dental restoratives. They are applicable for both drug delivery and tissue engineering. Advanced hydrogel systems, particularly when combined with injectable forms and programmable 3D bioprinting, can support precision medicine and restoration across diverse dental conditions [284], [285].

The performance of composite resins is largely influenced by embedded microcapsules, which govern self-healing and mechanical properties. Key limitations include the undetermined optimal microcapsule size, as too small a size limits functionality while too large a size compromises mechanical properties; the uncertain ideal shell thickness, which must withstand mixing yet rupture during self-healing; the potential formation of voids upon rupture, affecting mechanical integrity and surface smoothness; and the undefined microcapsule addition ratios, long-term stability, and shelf life. Addressing these factors is crucial for optimizing core systems, dosage, and self-healing efficiency.

High repair efficiency has been achieved for microencapsulated materials through system optimization. Nevertheless, challenges remain, including high costs, slow repair rates, and complex preparation processes. Future directions include: 1. Enhancing self-repair efficiency via highly active catalysts such as rare-earth trifluoromethanesulfonate and SbF${}_{5}$. 2. Developing new microencapsulation methods using hollow microcapsules, glass spheres, or nanotubes as carriers for highly active catalysts. 3. Expanding the application of click chemistry for self-healing materials, leveraging hydrophobic groups that react with epoxy, olefin, cyanate ester, and maleimide at room temperature. Future research should focus on greener, more stable, and environmentally friendly agents.

**Fig. 11:**
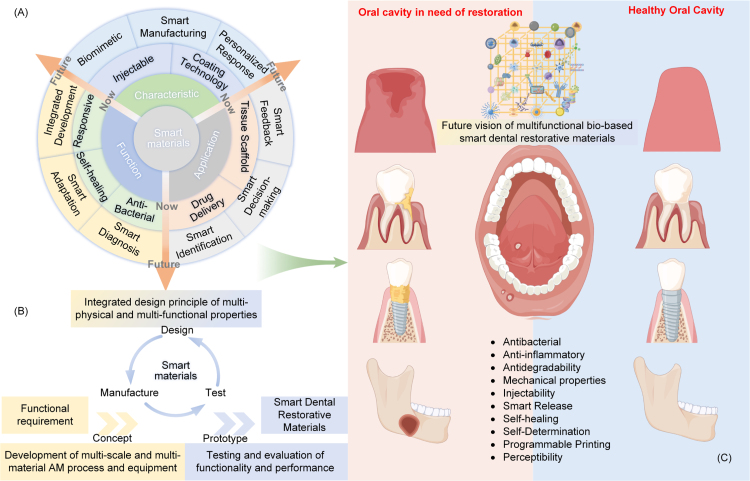
Future trends and system components of intelligent dental restorative materials.

In summary, research aims to develop smart dental restorative materials that retain the benefits of original self-restorative materials while adding functionalities such as self-healing and antimicrobial effects, achieving controlled, localized, and timed responses, and incorporating injectability, programmability, and regenerative capabilities. Fig. 11A illustrates current characteristics and potential development trends of smart dental restorative materials.

### 5.3 Commercialization of smart dental materials and current status of clinical applications

With the continuous development of smart materials, a variety of commercially available smart oral restorative materials have emerged. These materials are designed to address multiple challenges in oral restoration, including self-healing, antimicrobial activity, and remineralization. For example, 3M’s Filtek™ Supreme Ultra composite resin, with its superior antimicrobial and self-healing capabilities, is already widely used in clinical practice. However, while its short-term performance is promising, the sustainability of its self-repairing ability over long-term use requires further validation. Similarly, Dentsply Sirona’s SureFil™ SDR Flow exhibits favorable flow and remineralization properties, but its antimicrobial effect is limited, and its long-term efficacy in complex clinical conditions remains uncertain.

Nanomaterials, such as Grandio® So, demonstrate the potential of nanotechnology to enhance mechanical strength and antimicrobial properties, though issues of biocompatibility and cytotoxicity remain critical challenges. ThermoSense™, a thermosensitive hydrogel, offers excellent biocompatibility and tunable degradability, yet its mechanical stability remains a concern.

Looking forward, advancements in nanotechnology, 3D printing, and artificial intelligence (AI) are expected to drive breakthroughs in personalized treatment, precise drug release, and smart responsiveness. In particular, integrating nanotechnology with smart sensors could enable real-time monitoring of oral conditions and precise regulation of therapeutic agent release, enhancing both efficacy and long-term stability of restorative materials. These innovations promise more personalized and efficient clinical solutions in restorative dentistry.

### 5.4 Regulatory oversight and AI-assisted advancements in smart dental materials

Despite their technological promise, the clinical translation of smart dental restorative materials faces significant regulatory challenges. Agencies such as the US FDA and China CFDA/NMPA have issued guidelines for new dental materials; however, specific regulations for smart materials, including self-healing and responsive systems, remain under development.

For certain smart dental materials, such as self-repairing composite resins and nanocomposites, the FDA’s 510(k) pathway may apply, provided substantial equivalence to existing devices can be demonstrated [171]. For novel materials with no existing analogues, manufacturers may pursue the De Novo pathway, suitable for low- to medium-risk devices. High-risk materials, such as those containing active pharmaceutical ingredients or unique nanostructures, typically require PMA approval, which entails rigorous clinical evaluation and long-term safety assessment. Biocompatibility testing, referencing ISO 10993 standards, is essential before clinical application [157].

Similarly, CFDA/NMPA regulations distinguish approval pathways based on material risk. Low-risk restorative materials may follow fast-track approval, while innovative or active-ingredient-containing materials require clinical trial data to demonstrate safety and efficacy. Trials must assess physical properties, biosafety, durability, and performance within the oral cavity over extended periods. High-risk materials undergo more stringent evaluation, analogous to FDA PMA requirements.

**Fig. 12:**
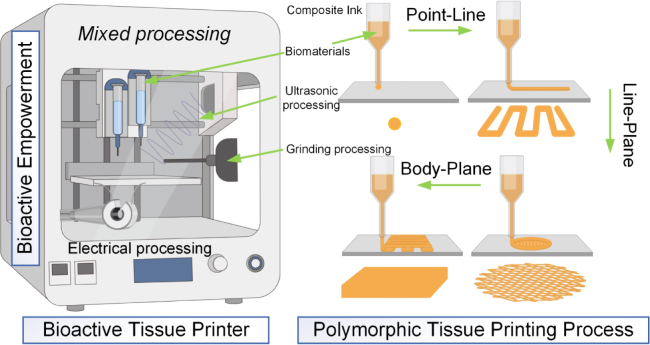
Hybrid machining process with additive manufacturing flow.

AI and machine learning (ML) are revolutionizing smart dental material development. Big data-driven material genomics approaches enable the optimization of formulations and reverse design of polymers and fillers, accelerating discovery. ML models can predict long-term material performance under complex oral conditions, including aging, mechanical stability, and biocompatibility, reducing experimental costs and time. Closed-loop discovery (CLD) workflows, integrating high-throughput experimentation with AI algorithms, facilitate automated design, validation, and optimization of materials, advancing intelligent. Future progress depends on high-quality, multimodal datasets covering chemical structure, physical properties, biological responses, and clinical performance. The integration of AI, high-throughput screening, and 3D printing may realize fully automated discovery, synthesis, and testing of smart dental materials, expediting translation from laboratory research to commercialization. AI-assisted development also supports the exploration of new smart materials, enhancement of existing composite resins and hydrogels, and optimization of performance in multi-stimulus environments, ultimately providing robust, personalized strategies for restorative dentistry, as shown in Fig. 12.

## 6 Conclusions

This review highlights the rapid progress and future potential of smart dental restorative materials, with a particular focus on composite resins and hydrogels. Compared with conventional materials, these novel systems provide enhanced self-healing, antibacterial, and remineralization functions, thereby addressing major challenges such as secondary caries, microleakage, and insufficient long-term stability of restorations. Current findings indicate that nano-enhanced fillers, optimized polymer networks, and advanced microcapsule-based delivery systems can significantly improve mechanical strength, fatigue resistance, and functional responsiveness under the complex oral environment. Hydrogels, particularly those compatible with minimally invasive surgical techniques and programmable 3D bioprinting, offer new avenues for tissue engineering and personalized therapy. These advances suggest that restorative materials will evolve from passive fillers to intelligent therapeutic platforms capable of dynamic adaptation to oral microenvironments. Nevertheless, important barriers remain, including the long-term durability of smart functions, biosafety of nanocomponents, and regulatory approval for clinical translation. The integration of cutting-edge technologies such as nanotechnology, artificial intelligence, and high-throughput screening is expected to accelerate material design, performance prediction, and clinical readiness. Future research should prioritize multimodal response capabilities, environmentally sustainable chemistries, and bioinspired functionalization to meet the increasing demands for precision, personalization, and longevity in restorative dentistry. In summary, smart restorative materials are poised to transform dental treatment paradigms. Through continuous optimization and validation, they will not only extend the service life of restorations but also pave the way toward safer, more effective, and patient-centered oral healthcare.

## 7 Abbreviations

**Table:** 

**Abbreviation**	**Full Term**

AAMP	Amphiphilic Antimicrobial Peptides
ACP	Amorphous Calcium Phosphate
AgNP	Silver Nanoparticles
Al${}_{2}$O${}_{3}$	Aluminum Oxide
AMPs	Antimicrobial Peptides
aPDT	Antimicrobial Photodynamic Therapy
CHX	Chlorhexidine
CS	Chitosan
DMAHDM	Dimethylaminododecyl Methacrylate
Fe${}_{3}$O${}_{4}$	Iron Oxide
GelMA	Gelatin Methacryloyl
GH_12_	ϵ-Polylysine
GIC	Glass Ionomer Cement
GMSCs	Gingival Mesenchymal Stem Cells
IAIs	Implant-Associated Infections
I-HAp	Ion-substituted Hydroxyapatite
MSCs	Mesenchymal Stem Cells
MTR	Metronidazole
NACP	Nano-Amorphous Calcium Phosphate
NACPs	Nanoactive Particles
NPC	Nanoparticle Carrier
NPs	Nanoparticles
PCL	Poly(ϵ-caprolactone)
PDA NPs	Polydopamine Nanoparticles
PDMA	Poly(dimethylacrylamide)
PEG	Polyethylene Glycol
pH	Potential of Hydrogen
SDF	Silver Diamine Fluoride
SilMA	Silk Proteins–Glycidyl Methacrylate
TiO${}_{2}$	Titanium Dioxide
ZIF${}_{8}$	Zeolite Imidazolate Framework 8
β-GP	β-Glycerophosphate
	

## CRediT authorship contribution statement

**Jianpeng Sun:** Writing – original draft, Supervision, Software, Project administration, Methodology, Funding acquisition, Data curation, Conceptualization. **Jingang Jiang:** Visualization, Validation, Supervision, Software, Resources, Project administration, Methodology, Investigation, Funding acquisition, Conceptualization. **Zhiyuan Huang:** Writing – review & editing, Validation, Supervision, Software, Methodology, Funding acquisition, Formal analysis, Data curation, Conceptualization. **Xuefeng Ma:** Visualization, Validation, Supervision, Project administration, Methodology, Formal analysis, Data curation, Conceptualization. **Tao Shen:** Visualization, Validation, Supervision, Software, Resources, Methodology, Data curation, Conceptualization. **Jie Pan:** Visualization, Validation, Supervision, Software, Resources, Methodology, Funding acquisition, Data curation. **Zhuming Bi:** Writing – review & editing, Visualization, Validation, Supervision, Software, Resources, Funding acquisition, Formal analysis, Conceptualization.

## Declaration of competing interest

We declare that they have no known competing financial interests or personal relationships that could have appeared to influence the work reported in this paper.

## Data Availability

Data will be made available on request.
